# Comparative Analysis of Proteome and Transcriptome Variation in Mouse

**DOI:** 10.1371/journal.pgen.1001393

**Published:** 2011-06-09

**Authors:** Anatole Ghazalpour, Brian Bennett, Vladislav A. Petyuk, Luz Orozco, Raffi Hagopian, Imran N. Mungrue, Charles R. Farber, Janet Sinsheimer, Hyun M. Kang, Nicholas Furlotte, Christopher C. Park, Ping-Zi Wen, Heather Brewer, Karl Weitz, David G. Camp, Calvin Pan, Roumyana Yordanova, Isaac Neuhaus, Charles Tilford, Nathan Siemers, Peter Gargalovic, Eleazar Eskin, Todd Kirchgessner, Desmond J. Smith, Richard D. Smith, Aldons J. Lusis

**Affiliations:** 1 Department of Medicine/Division of Cardiology, David Geffen School of Medicine, University of California Los Angeles, Los Angeles, California, United States of America; 2 Biological Sciences Division and Environmental Molecular Sciences Laboratory, Pacific Northwest National Laboratory, Richland, Washington, United States of America; 3 Department of Human Genetics, University of California Los Angeles, Los Angeles, California, United States of America; 4 Department of Medicine, Department of Biochemistry and Molecular Genetics, and Center for Public Health Genomics, University of Virginia, Charlottesville, Virginia, United States of America; 5 Biostatistics Department, University of Michigan Ann Arbor, Ann Arbor, Michigan, United States of America; 6 Department of Computer Science, University of California Los Angeles, Los Angeles, California, United States of America; 7 Department of Molecular and Medical Pharmacology, David Geffen School of Medicine, University of California Los Angeles, Los Angeles, California, United States of America; 8 Department of Applied Genomics, Bristol-Myers Squibb, Princeton, New Jersey, United States of America; 9 Department of Atherosclerosis Drug Discovery, Bristol-Myers Squibb, Princeton, New Jersey, United States of America; 10 Molecular Biology Institute, University of California Los Angeles, Los Angeles, California, United States of America; 11 Department of Microbiology, Immunology, and Molecular Genetics, University of California Los Angeles, Los Angeles, California, United States of America; Stanford University School of Medicine, United States of America

## Abstract

The relationships between the levels of transcripts and the levels of the proteins they encode have not been examined comprehensively in mammals, although previous work in plants and yeast suggest a surprisingly modest correlation. We have examined this issue using a genetic approach in which natural variations were used to perturb both transcript levels and protein levels among inbred strains of mice. We quantified over 5,000 peptides and over 22,000 transcripts in livers of 97 inbred and recombinant inbred strains and focused on the 7,185 most heritable transcripts and 486 most reliable proteins. The transcript levels were quantified by microarray analysis in three replicates and the proteins were quantified by Liquid Chromatography–Mass Spectrometry using O(18)-reference-based isotope labeling approach. We show that the levels of transcripts and proteins correlate significantly for only about half of the genes tested, with an average correlation of 0.27, and the correlations of transcripts and proteins varied depending on the cellular location and biological function of the gene. We examined technical and biological factors that could contribute to the modest correlation. For example, differential splicing clearly affects the analyses for certain genes; but, based on deep sequencing, this does not substantially contribute to the overall estimate of the correlation. We also employed genome-wide association analyses to map loci controlling both transcript and protein levels. Surprisingly, little overlap was observed between the protein- and transcript-mapped loci. We have typed numerous clinically relevant traits among the strains, including adiposity, lipoprotein levels, and tissue parameters. Using correlation analysis, we found that a low number of clinical trait relationships are preserved between the protein and mRNA gene products and that the majority of such relationships are specific to either the protein levels or transcript levels. Surprisingly, transcript levels were more strongly correlated with clinical traits than protein levels. In light of the widespread use of high-throughput technologies in both clinical and basic research, the results presented have practical as well as basic implications.

## Author Summary

An old dogma in biology states that, in every cell, the flow of biological information is from DNA to RNA to proteins and that the latter act as a working force to determine the organism's phenotype. This model predicts that changes in DNA that affect the clinical phenotype should also similarly change the cellular levels of RNA and protein levels. In this report, we test this prediction by looking at the concordance between DNA variation in population of mouse inbred strains, the RNA and protein variation in the liver tissue of these mice, and variation in metabolic phenotypes. We show that the relationship between various biological traits is not simple and that there is relatively little concordance of RNA levels and the corresponding protein levels in response to DNA perturbations. In addition, we also find that, surprisingly, metabolic traits correlate better to RNA levels than to protein levels. In light of current efforts in searching for the molecular bases of disease susceptibility in humans, our findings highlight the complexity of information flow that underlies clinical outcomes.

## Introduction

An underlying assumption in many biological studies is the concordance of transcript and protein levels during the flow of information from DNA to phenotype. Clearly, protein levels are greatly influenced by post-translational processing and inherent variations in stability but, in general, it is assumed that perturbations of transcript levels are substantially correlated with protein levels. The extent to which this occurs, however, remains poorly understood and understanding the relationships across scales, from DNA to phenotype, has both practical and basic implications. For example, “genetical genomics” studies examine transcript levels as a function of genetic variation and use this information to construct models, such as interaction networks, to explain complex phenotypes [1]–[8]. Systems based approaches, in particular, have relied heavily on transcriptome data [9].

Concordance of protein and transcript levels has been studied in yeast and plants. A recent comparative study in a yeast segregating population showed that there is a significant but modest correlation between transcript and protein levels [10]. Moreover, this report also found that, in general, loci that influence protein abundance are different from those affecting transcript abundance. A similar comparative analysis of molecular phenotype mapping in Arabidopsis [11] was reported subsequently. In this report the authors investigated the commonality of hotspot loci (defined as loci affecting a large number of traits within each biological class) across various biological scales and observed a general theme consistent with the phenotypic buffering of perturbations affecting molecular phenotypes as one looks to scales further away from the DNA variation (e.g. proteome vs transcriptome). Both of these reports emphasize the value gained from bringing together information from various biological scales, as each dataset will add new information to the phenotypic effect of DNA variation.

We now report global analysis of transcript-protein relationships in mice using a genetic approach involving thousands of naturally occurring perturbations. For this, we have utilized a recently developed panel of permanent inbred strains of mice, termed the Hybrid Mouse Diversity Panel (HMDP), that allows high resolution mapping of complex traits [12]. We chose to examine protein and transcript levels in liver given the importance of the organ in metabolic traits relevant to disease.

## Results

### Study design

**Figure 1:**
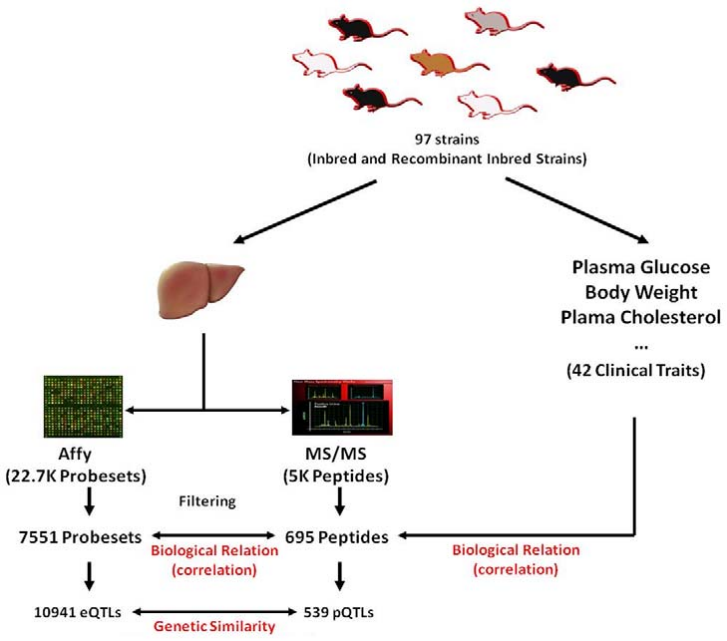
A schematic representation of the experimental design. 97 inbred and recombinant inbred strains in the HMDP panel were utilized to study the relationships between transcripts, proteins, and clinical traits. The relationships between proteins and transcripts were assessed at the biological level by the overall correlation across datasets, and at the genetic level by comparing the genome-wide association profiles of the two datasets. The biological relationship between the transcripts and proteins was also assessed in the context of the physiological phenotypes by relating these two datasets to the 42 clinical traits measured in the HMDP panel.

The experimental design of our study is depicted in Figure 1. To study the relationship between transcript and protein levels globally, we examined 97 inbred strains of mice of the HMDP representing a wide range of genetic diversity, including ∼11,000,000 single nucleotide polymorphisms as well as copy number variations [13], [14]. As we have shown previously, this population includes thousands of expression quantitative trait loci (eQTL) that can be mapped in the population using association analysis with correction for population structure using a mixed model algorithm [12]. The resolution achieved in this way is, on average, one to two orders of magnitude narrower than that using linkage analysis [12]. Livers from the 97 strains were quantitatively analyzed for global transcript levels using the Affymetrix HT-MG-430A platform and for protein levels using LC-MS employing AMT tag approach for identification and ^16^O/^18^O labeling for quantification [12], [15]. In the latter, each individually processed and unlabeled sample is spiked with the ^18^O labeled “universal” reference pool (i.e. the pool made from mixing together the same amount of isolated proteins from all samples) providing an internal standard for accurate measurement of protein abundance across biological samples. This dual-quantification, which combines the label-free and isotope labeling techniques, has been shown to be significantly superior over label-free methods in terms of quantification precision [15] and offers a simple, robust, and a more precise alternative to other proteomic techniques for studying variations in protein levels across large biological samples. In the LC-MS dataset, we also included 10 technical replicates from the C57BL/6J strain to measure the reproducibility of the sample preparation and technology which we describe in detail below.

### Peptide and microarray data quantification and quality

One technical issue for proteomic analysis relates to how peptides with non-synonymous coding SNPs would present themselves in the correlation and association analyses. In order to annotate peptides detected by the tandem LC- MS/MS fragmentation patterns, the mass spectra are matched against a pre-established known reference database. In our case this database, which was built from the pool of all the inbred strains in the HMDP panel, was created by annotating the peptides against the reference sequence (C57BL/6J strain) followed by filtering out those peptides which have non-synonymous coding SNPs documented in public database for any of the HMDP strains. As a result of this preprocessing step, the peptides identified by LC-MS were limited to those that did not contain known non-synonymous SNPs in their amino acid sequences.

**Figure 2:**
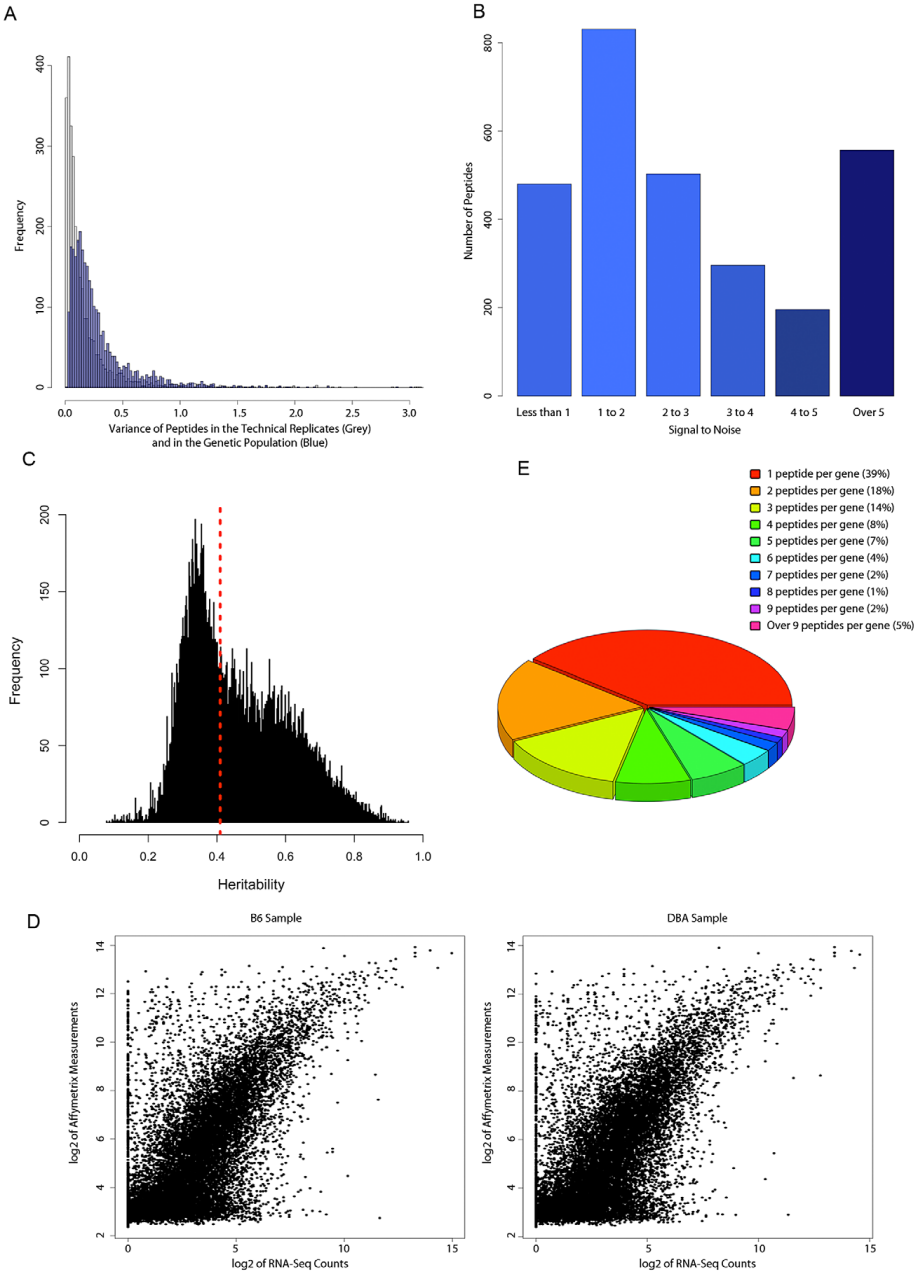
Proteome and transcriptome data quality. A) Reliability of peptide measurement in LC-MS. The distribution of variance among the technical replicates in the LC-MS data (grey plot) and in the HMDP population (blue plot). B) The frequency of peptides with varying amount as defined by the “signal to noise” ratio. C) Distribution of heritability (fraction of total variance attributed to genetics) in the transcript dataset. The dashed line depicts the significant heritability estimates (p-value<0.05) D) Comparison of Affymetrix data with the Next Generation Sequencing data. E) Number of peptides per gene in the filtered peptide dataset.

From the original 5363 peptides measured, we selected peptides that a) had less than 50% missing measurements in the whole population, b) had no internal lysine or arginine, and c) aligned uniquely to one Ensembl gene. Fifty four percent of peptides (2893 peptides) passed these initial selection criteria. To assess the quality of the measurements, we investigated the amount of technical noise in the peptides selected. Having the control technical replicates allowed us to measure the reproducibility of the LC-MS measurement and assess whether the variation in the levels of the selected peptides in the HMDP population was due to technical or genetic variation. The distribution of the variance in the control mice and in the HMDP panel are shown in Figure 2A (the blue histogram). The mean and median across the ten replicates were 0.19 and 0.08 (the grey histogram), respectively, suggesting that, for most peptides, the measurements were robust. In contrast, the distribution of the variance was much broader in the genetic population where the mean and median of variances across all the peptides were 0.2 and 0.3 respectively (Figure 2A, the blue histogram).

The relationship between RNA levels and peptide levels across the HMDP genetic perturbations would be a function of the genetic variation in the peptide levels as well as the degree of nongenetic/technical variations in peptide quantification. Thus, we defined a “signal to noise” measure for each peptide as the ratio of the total variance in the HMDP over the variance in the ten replicates. The variance in the ten replicates would be due to nongenetic biologic variance as well as technical variance (herein termed “noise”) while the total HMDP variance would include genetic variance, nongenetic biologic variance, and technical variance. Accordingly, a large value of signal to noise could either mean large genetic variation, small nongenetic variation, or both. Conversely, a smaller value for signal to noise would either mean small genetic variation, large nongenetic variation, or both. As can be seen in Figure 2B, signal to noise ratios varied significantly across different peptides.

We complemented the LC/MS studies for a small set of proteins (11) by performing immunoblot quantitation in 9 of the HMDP strains. Over half of the peptides exhibited significant discrepancies in relative levels using the two methods and those with small “signal to noise” ratios (small genetic variation and/or large noise component) exhibited reduced correlations with the immunoblotting results (p-value = 3.3×10^−5^). Although immunoblotting is semi-quantitative, these results suggest that peptides with a large signal to noise ratio will provide the best estimates of the true relationship between RNA levels and protein levels. (Immunoblot results are presented in Table S1, Table S2, and Figure S1).

Transcript levels in inbred stains were measured by profiling three mice for each strain, using Affymetrix MOE430A platform, and taking the average of expression over the three biological replicates. This design provided us with an opportunity to a) better estimate the “true” values for the mRNA levels in each strain and b) estimate the heritability of each probeset across the HMDP population. The distribution of heritability estimates is shown in Figure 2C. Consistent with previous reports [16], we detected a broad spectrum of heritability estimates for the transcript levels ranging from 0.07 to 0.95. Using ANOVA, we assigned significance to the heritability values obtained for each probeset and found that for as many as 50% of the probesets (11248 probesets), there was a significant (p-value<0.05 for strain term) genetic component affecting the transcript levels.

Previous reports have documented conflicting results about the reproducibility measurements generated by microarray platforms [17]–[21]. To investigate this in our dataset, we compared the expression levels measured by the Affymetrix microarray to the expression levels measured by next generation sequencing (NGS) in small subset of inbred strains. Using Illumina's Genome Analyzer we profiled the liver transcriptome of one C57BL/6J and one DBA/2J mouse and generated ∼17,000,000 sequences for the fragmented mRNAs for each strain. Using Tophat [22] and Cufflink [23] algorithms, we were able to uniquely align and count 4,800,000 and 7,000,000 sequences for C57BL/6J and DBA/2J respectively (See Materials and Methods). After sequence alignment and quantification of the read counts, we compared the transcriptome of the each strain against the microarray data generated by the Affymetrix MOE430a platform (Figure 2D). This comparison revealed a high concordance between the data obtained from two technologies (Spearman correlation coefficient of 0.69 for both C57BL/6J and DBA/2 samples). These results were similar to a previous cross-platform comparative study [24] and indicated that, for most transcripts, microarray data produce a highly reliable estimate of transcript levels.

Based on the results reported above, in order to enrich for high quality data and provide a better estimate of true relationships between transcript and protein data, we focused on transcript and peptide data with significant genetic and biological variation. For peptide data, we used the signal to noise ratio parameter to further filter the noisy peptides from our dataset. The cutoff we chose for filtering peptides was a signal to noise ratio of 2. After removing the “noisy” measurements, we were left with 1543 peptides (see Dataset S1 for the expression and Dataset S2 for the annotation of the peptides). These 1543 peptides represented 486 Ensembl Genes from which 39% were represented by only one peptide and the remaining 60% were represented by two or more peptides (Figure 2E). The most abundant number of peptides (>20 peptides per gene) was found for 2 genes, *aldehyde dehydrogenase 1 family*, *member L1 (Aldh1l1)*, and *carbamoyl-phosphate synthetase 1 (Cps1)*. For the transcript data, we focused only on those transcripts that had a) a significant genetic component underlying their variation (heritability p-value<0.05), and b) unambiguous annotation in the Ensembl database. To comprehensively compare the transcripts and peptides, we also included those probesets that were annotated as the same Ensembl gene/transcript as one of the peptides in the protein data. This resulted in the total of 9896 probesets (representing 7185 Ensembl genes) from the initial 22670 probesets.

### Proteome and transcriptome representation

To investigate the range of gene products present in the filtered datasets, we generated a separate list of “GO Slim” terms for each of the three major GO categories (Cellular Compartment or “CC”, Molecular Function or “MF”, and Biological Process or “BP”) and used the “GO Term Mapper” website (http://go.princeton.edu/cgi-bin/GOTermMapper) to classify and count the number of proteins and transcripts in each of the 3 major GO categories (Table S3). We also compared our results to the background set (all genes annotated by Mouse Genome Informatics) and using Fisher's Exact test calculated the degree of enrichment or underrepresentation for each GO class. Figure S2 depicts the results of this analysis for the Cellular Compartment GO terms. While the proteome and transcriptome datasets represent a wide range of gene products present in various cellular compartments, the compartments are not equally represented. In the protein data, mitochondrial genes were overwhelmingly the largest set and in the transcript data the nuclear compartment was at the top of the list for enriched CC GO terms. The enrichment analysis showed that for both protein and transcript datasets the majority of the GO terms tested were significantly over and under-represented and these differences were more pronounced for the protein data (Table S3). A likely explanation for this observation is that the LC-MS analysis of the liver provides a biased sampling of the proteome data (due to the abundance and/or cellular location of the protein). The significant differences between the transcript data and the background set may be partially explained by the bias introduced in the design of the Affymetrix microarray. Alternatively, since the transcript data by the virtue of filtering represent the significantly heritable subset of all transcripts present on the Affymetrix chip, one could postulate that in some cellular compartments or cellular processes transcripts are more or less likely to exhibit common genetic variation.

### Modest concordance between transcript and protein levels

**Figure 3:**
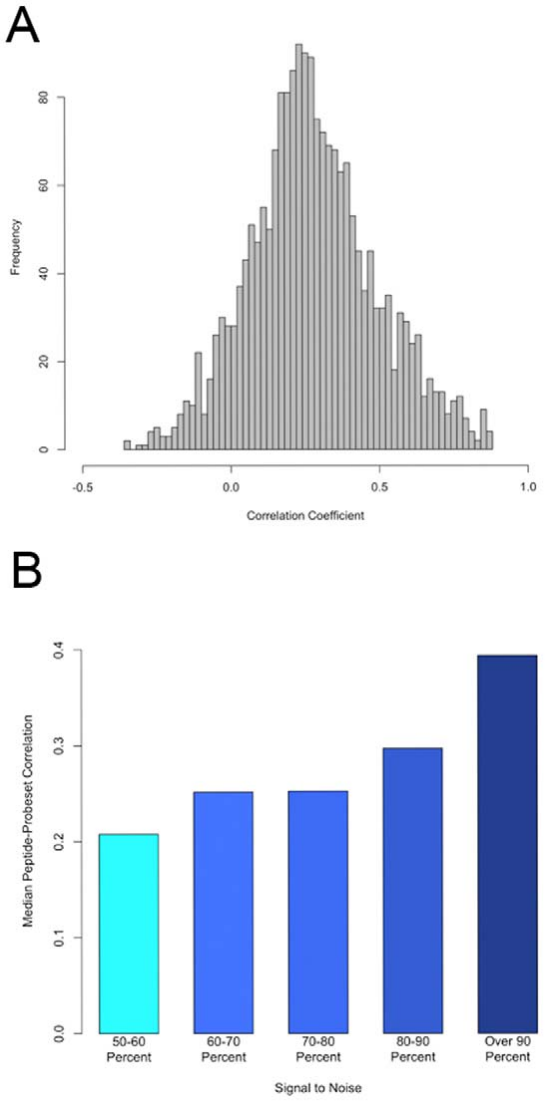
Relationships between protein levels and transcript levels. A) Histogram of correlation coefficients computed peptides and probesets representing the same gene. The median correlation coefficient is 0.27. B) Classification of correlations between probeset-peptides based on signal to noise ratio in the peptide data (larger signal to noise depicts less technical variation in the peptide measurement).

We next examined the degree of concordance between the transcript and protein levels. For this, we compared the transcript and peptide measurements for every peptide-probeset pair that mapped to the same Ensembl gene. This “gene-level” analysis included 2010 peptide-probeset pairs (1342 peptides and 607 probesets) representing 396 Ensembl genes. Figure 3A shows the correlation coefficient distribution for these 2010 peptide-probeset pairs. Highly significant positive correlation (p-value<1e-06, r>0.46) between RNA and protein was found for 21% of the genes (85 out of 396) and ∼15% of the peptide-probeset pairs (291 out of 2010). The most significant correlation (r = 0.87) was found for the glyoxalase 1 gene (*Glo1*) where the peptide and transcript of this gene correlated (Figure S3). Overall, we found that the relationship between mRNA and protein levels was modest (mean r = 0.27) and for 39% of the pairs (761 of 2010) the mRNA and protein levels did not correlate significantly at the nominal 0.05 p-value threshold. Our estimate of average correlation between mRNA and protein was slightly higher than those reported in other organisms, perhaps due to recent improvements in the LC-MS technology and/or statistical power.

Next, we examined if the amount of technical noise and/or lack of genetic variation could explain the modest correlation between mRNA and peptide data. For this we classified each peptide based on the signal to noise ratio (defined earlier) and looked at the median correlation between mRNA and peptides within each group. As shown in Figure 3B, we found that as the ratio of signal to noise increases so does the correlation between the mRNA and peptide levels of the gene. In fact the median correlation for the least noisy group, comprising peptides with signal to noise ratio >90%, was twice as large as the noisiest group of peptides (peptides with signal to noise ratio <60%). These results suggests that the modest correlation between peptides and mRNA observed in our study is partially due to either the presence of significant nongenetic variation or small genetic variation in some proteins.

### Alternative splicing is not a significant contributing factor to the overall modest correlation of transcript and protein levels

**Figure 4:**
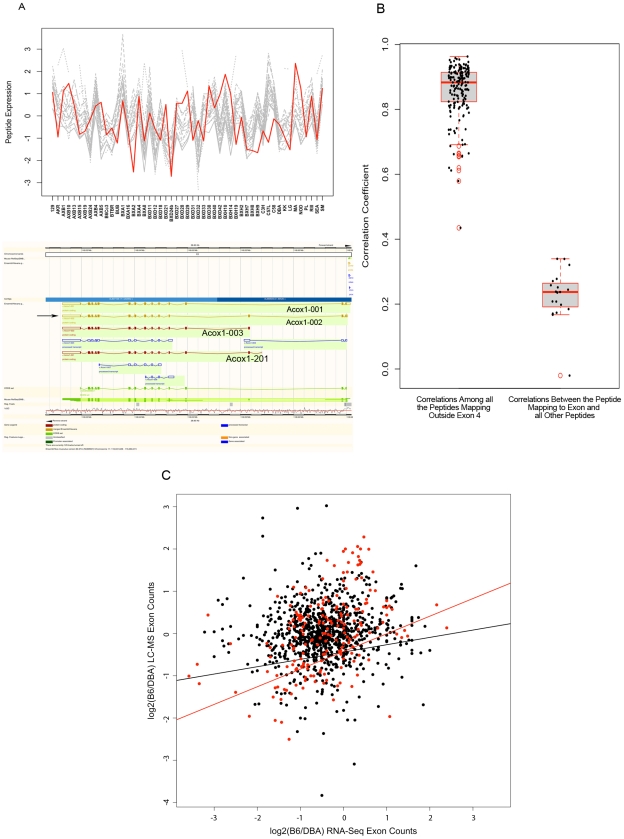
Isoform-specific analysis of peptide data. A) An example of differential regulation of isoforms detected in the LC-MS data. Top panel, comparison of similarity in expression variation of 20 peptides measured for Acox1. Grey plots illustrate the expression variation among inbred mice for 19 peptides which represent all four Acox1 isoforms. Red plot illustrated the expression profile of the peptide representing the isoforms skipping exon 4. Bottom panel, Ensembl genome browser's schematic representation of four Acox1 isoforms. Arrow points to Acox1-002 isoform which skips exon 4. B) Concordance between Acox1 peptides. The left boxplot depicts correlations among peptides that include Acox1-002 isoform. The right boxplot depicts correlations between the peptide mapping to exon 4 and all other peptides. The scatter points overlaid on each boxplot represent the pair-wise correlation values. C) Exon level analysis of peptide measurements by LC-MS and transcript measurements as measured by NGS in the livers of the B6 and DBA inbred strains. The black dots depict the relationships examined by comparing peptide data to microarray data and the red dots represent the highly significant relations found by peptide comparison with the microarray data. The lines depict the best fit as predicted by linear regression (black line = regression of all peptides, red line = regression of highly significant peptides).

Aside from lack of genetic variation in peptides, another plausible explanation for the lack of high correlation between peptides and probesets could be the analytic approach chosen to calculate correlations. In our study, we estimated the relationship between mRNA and proteins by examining the correlations between pairs of peptides and probesets that were annotated to the same gene without considering the isoform information for that gene. The choice of analytic approach presented here was mainly due to the limitation of the technology we used to measure the transcript levels. The probesets on the Affymetrix microarrays are designed to hybridize mainly to the transcripts 3′ end. Such design will fail to accurately measure the levels of isoforms which are identical at the 3′ end but are differentially regulated at the transcript level. The inability to measure isoform specific expression can clearly impact the mRNA-protein correlation results for certain peptides which represent specific isoforms as LC-MS data may include peptides unique to a gene's isoform. Figure 4A and 4B illustrate an example of differential isoform regulation identified in the LC-MS data. Acox1 (*acyl-Coenzyme A oxidase 1*, *palmitoyl*) is a peroxisomal gene involved in fatty acid beta-oxidation pathway and metabolism of very long chain fatty acids, and its deficiency causes pseudoneonatal adrenoleukodystrophy [25] in humans. This gene produces four protein-coding products (Acox1-001, Acox1-002, Acox1-003, and Acox1-201 as denoted in Ensembl genome browser) shown in Figure 4A (bottom panel). All isoforms except for “Acox1-002” include exon 4 of this gene. In LC-MS data, 20 peptides were measured for this protein. One of these 20 peptides (“GHPEPLDLHLGMFLPTLLHQATEEQQER”) maps to the exon 4 sequence of this gene, thus, does not represent the “Acox1-002” isoform which skips this exon. Examining the expression profile and correlation of these 20 peptides revealed that all peptides representing “Acox1-002” isoform are highly intercorrelated (mean r = 0.86, Figure 4B) and exhibit a similar expression profile (Figure 4A, top panel), but none have either similarity in expression profile or significant correlation with the peptide mapping to the exon 4 which is skipped in Acox1-002 isoform (mean r = 0.23, Figure 4A top panel, and 4B). This suggests that Acox1-002 isoform (with the skipped exon 4) is the main isoform underlying the significant correlation among 19 of the 20 peptides identified by LC-MS in our genetic population. This example illustrates that the LC-MS data contain information on differential regulation of isoforms, in contrast to the microarray data.

To investigate if our inability to measure isoform specific expression by microarrays could explain the lack of concordance between mRNA and peptides, we utilized the next generation sequencing data generated for the two inbred strains described earlier. This dataset provided us with an opportunity to examine the transcript level expression of the exons measured by NGS with the protein level expression of exons measured by the LC-MS. To investigate this, for each peptide we calculated the count of exons in RNA-Seq data for two strains. We then compared the DBA to B6 ratio of each exon in the peptide data to the DBA to B6 ratio of normalized sequence counts (reported in FPKM units) in the RNA-Seq data. The results of these comparisons are shown in Figure 4C. Similar to the gene-level analysis, the exon level analysis of all the filtered data also suggested a modest relationship between the exon counts in the mRNA data vs exon levels in the LC-MS data (r^2^ = 0.02). This global analysis provided no support for the presence of differential splicing/isoform regulation as being a significant factor in the mRNA and protein overall relationships observed between LC-MS and microarray data. The relationship between RNA-Seq data and LC-MS peptide data is particularly strong (r = 0.42) for those peptides exhibiting a strong correlation (r>0.5) with microarray data (Figure 4C).

In an alternate approach to study the effect of differential splicing on the correlation pattern, we examined our LC-MS data at the isoform level and compared the results to the gene level analysis. For this, we grouped various peptides of each protein into unique and mutually exclusive clusters of known isoforms as defined by the Ensembl database. In this classification we allowed peptides to only represent one Ensembl protein ID and excluded any peptide which matched with two or more Ensembl proteins. Focusing on clusters with at least two peptides assigned to a cluster, we calculated the within cluster correlation of of peptides and compared the average within-cluster correlation to the average correlation of peptides at the gene level analysis. The average correlation of peptides at the gene level analysis was estimated at 0.47. In comparison, the average within cluster correlation of peptides representing the same isoforms was estimated to be 0.52. Combined with the NGS analysis described earlier, the small and nonsignificant increase in the peptide concordance after taking into account the isoform membership provides little support for differential regulation of splicing/isoform expression as a significant factor underlying the observed modest correlation between transcripts and proteins.

### Differential relationships of proteins and transcripts to clinical traits

**Figure 5:**
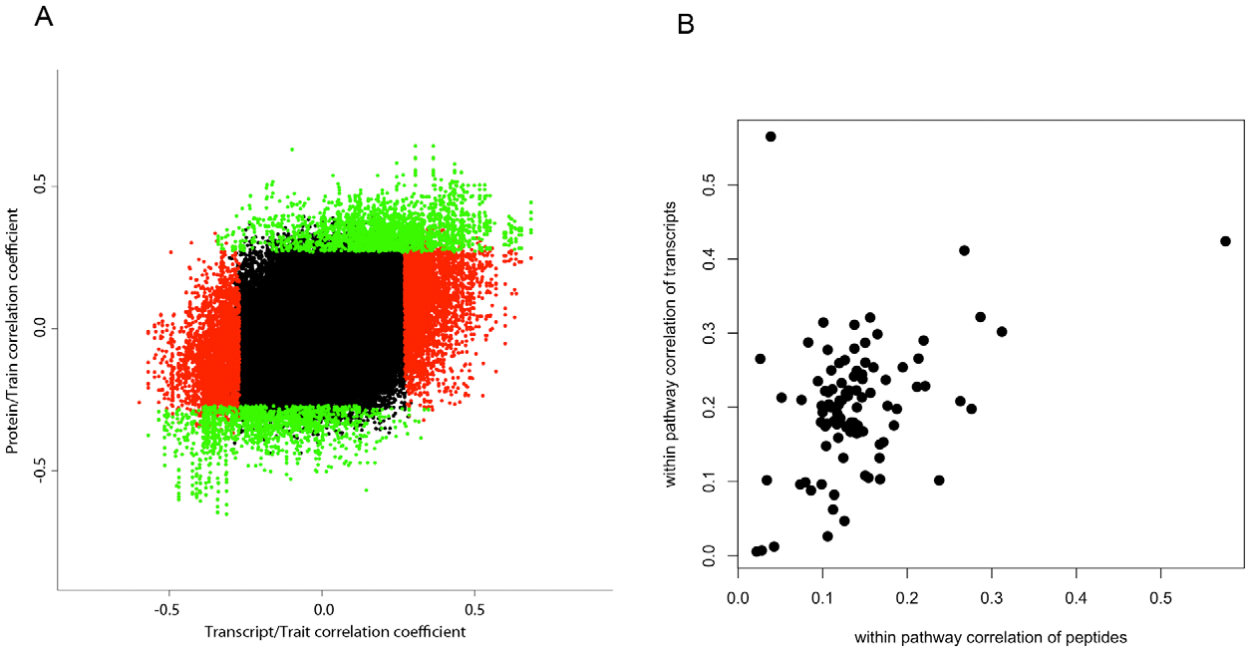
Relationships between the peptide data and transcript data with clinical traits and biological pathways. A) Correlations of transcriptome and proteome with clinical traits. A scatter plot of correlation coefficients between 607 probesets and 1343 peptides with 42 clinical traits (peptide-trait correlations are plotted on the x-axis and probeset-trait correlations are plotted on the y-axis). Red points are those correlations which were significant for transcripts only, green points are those correlations which were significant for protein data only and black points are those which were not significant in either of the two datasets. B) Concordance of transcripts and proteins in 115 KEGG biological pathways.

In light of the modest correlation observed between the transcript and protein pairs, we examined the relationship of each of these two datasets with clinical traits. In our HMDP panel, we have previously measured a set of 42, some interrelated, metabolic traits (see Materials and Methods). In this analysis, in order to make a direct comparison across the two datasets, we once again focused on the 396 genes for which we had at least one peptide and one transcript measurement. At the 5% false discovery rate, we observed that three quarters of probesets (457 from the total 607) significantly correlated with at least one of the clinical traits. In contrast, at the same false discovery rate, only 28% of the total peptides (380 out of 1342) showed significant correlation with at least one of the 42 phenotypes. Despite the fact that the starting number of peptides was twice the number of probesets (1342 and 607), the total number of significant correlations for the peptides was only about half the number found for the probesets (2206 vs 1107). The same biased pattern was also observed at other statistical thresholds as shown in Table 1. In addition to probeset-pair analysis, we also carried a similar analysis at the gene level to estimate what fraction of starting genes (396 total genes) a) exhibit consistent relationship with clinical traits both at the transcript level and the protein level b) exhibit trait relationships unique to either of the two molecular phenotypes. From the 396 genes, 325 genes had at least one significant correlation at the 5%FDR with clinical phenotypes and 162 had at least one significant correlation with phenotypes at the protein level (Table 1). At the transcript level, the total number of significant correlations amounted to 1781 vs 556 found at the protein level. From these, 234 relations were found to be common for transcript and protein of the same genes and 1547 were unique to transcripts only (Figure 5A). Despite this overwhelming bias toward better correlation of transcripts, we also found 322 unique relations at the protein level (Table 1 and Figure 5A). Altogether, about half the significant protein-trait correlations also exhibited transcript-trait correlations, but only 15% of the significant transcript-trait correlations exhibited corresponding protein-trait correlations.

**Table 1:** Relationship of PT-pairs with clinical traits.

	# Trait Correlations Unique to Proteins	# Trait Correlations Unique to Transcripts	# Trait Correlations Shared Between Transcripts and Proteins	# Proteins with Significant Correlation (%)	# Transcripts with Significant Correlation (%)
0.1% FDR	35	272	17	24 (6)	122 (31)
1% FDR	93	704	71	64 (16)	217 (55)
5% FDR	322	1547	234	162 (41)	325 (82)

### Relationships as a function of Gene Ontology categories and KEGG pathways

We sought to examine whether the concordance between protein and transcript data was dependent on the biological function and/or cellular location of the gene product. For this we restricted the list of genes within each of the 3 major GO_slim terms described earlier to the 396 genes for which we had at least one probeset and one peptide measured. We then defined the average relationship between protein and transcript products of the genes within each GO category by computing the correlation between the gene products and taking the average of these correlations. The three panels in Figure S4 show the average correlations of the transcript and protein product of the genes grouped by their assigned GO categories. Striking differences in the concordance between proteins and transcripts across some of the GO categories were observed. For example, for Cellular Compartment GO terms (CC), we found that peroxisomal and ER genes have on average a better correlation between protein and transcript products than other cellular compartments. We also found that for some of the GO categories the similarity between protein and transcript levels was almost non-existent (for example in BP the “cell growth” class, Figure S4C). To assess the significance of these observations, for each GO class we created 100,000 bootstrap datasets (each the size of the number of genes assigned to the respective GO category) containing correlation coefficient p-values randomly selected from the pool of peptide-probeset correlation p-values. We then assessed the significance of observed averaged correlation p-value for each class by comparing it to the distribution of the averaged p-values in the bootstrapped dataset (Table S4). In some GO groups, we found a class of genes for which the relationship between the transcript level and protein level is significantly better than for other GO groups. We also found GO classes in which the transcript levels and protein levels of the genes were significantly discordant (i.e. the relationship between protein and transcript was significantly less than what would be expected by chance). For example, in MF we found that genes classified as having a role in “electron carrier activity” had a strong relationship among the protein and transcript levels (p-value = 8.9e-03) and this relationship is significantly compromised for genes with “transporter activity” (p-value = 4e-05). Another example of discordant group was genes involved in the translation process (p-value<1e-05). Interestingly, the “translation” category has been proposed recently to be involved in phenotypic buffering in a yeast genetic interaction network. Overall, these results indicate that cellular compartments and biological processes vary in the degree to which the linear relationships between transcript levels and their protein products are conserved.

We also examined the level of concordance among transcripts and proteins of genes that are members of the same biological pathway. For this, we focused on 212 biological pathways on the KEGG website (http://www.genome.jp/kegg/). We annotated the peptides and probesets according to their pathway membership as determined by their Ensembl gene IDs. Ninety nine out of 212 pathways contained genes for which we had both more than one transcript and more than one protein measured. Focusing on these 99 pathways, we then performed the following three correlation analyses: 1) correlation between peptides belonging to the same pathway, 2) correlation between probesets belonging to the same pathway, 3) correlation between probesets and peptides belonging to the same pathway. Comparing the results of these three analyses suggests that overall within-transcript correlation of biological pathway genes is higher than within-protein correlations (0.20 vs 0.14 mean Spearman correlation coefficients) (Figure 5B), and transcript-protein correlations are the weakest of all (mean Spearman correlation coefficient of 0.11). From the 99 pathways, 79 pathways had better between-transcript correlations than between-protein correlations and 20 had better between-protein correlations. We also observed that for most pathways when there was good concordance between the transcripts there was also good concordance between the peptides of that pathway (r = 0.39, Figure 5B).

### Modest concordance of genetic loci controlling transcript and protein levels

Next, we examined the genetic loci regulating protein and transcript levels. It is known that the presence of SNP within probe sequence can affect hybridization of the mRNA [26], leading to both type-I and type-II errors in the genomewide association analysis. In our Affymetrix dataset, as expected, we also observed a significant effect of SNPs on genomewide association results for fraction of the probes, as judged by comparing the significance level for local eQTLs between probesets before and after masking of probes containing publicly available SNPs (see Text S1 and Table S5 for details, and Dataset S3 for the list of probes which were masked from each probeset due to the presence of SNP). To minimize this technical artifact, we removed all the SNP-containing probes from their corresponding probeset before normalization of the data and eliminated all the probesets which contained 8 or more probes with SNPs (∼300 probesets fell in this category). Therefore, all the data reported below were generated from masked probesets.

**Figure 6:**
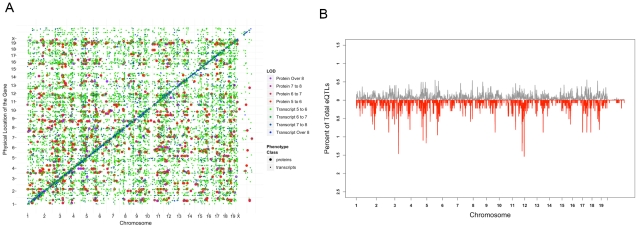
Global analyses of proteome and transcriptome genetic regulation. A) Global eQTL profile for the 14463 eQTLs and 1368 pQTLs superimposed on each other. In this plot, larger dots represent protein association and smaller dots represent transcript association. The diagonal line with strong association depicts the local eQTLs and pQTLs and each off-diagonal dot depicts the location of distant eQTLs and pQTLs. B) eQTL landscape for protein and transcript data. For each dataset, the genome was divided into 2 Mb bins and the number of eQTLs (grey) and pQTLs (red) were counted separately in each bin as the windows were slid every 50 kb. The frequency of eQTLs and pQTLs in each window are plotted as the fraction of total significant associations (14463 for transcripts and 1368 for proteins).

We performed genomewide association on both transcriptome and proteome datasets using 95,854 SNPs with minor allele frequencies greater than 10% obtained from the Broad Institute (http://www.broadinstitute.org/mouse/hapmap) and Wellcome Trust Center (WTCHG) (see Materials and Methods for details). To account for the population structure and genetic relatedness among strains in the genome-wide association mapping, we utilized the Efficient Mixed Modeling Algorithm (EMMA) [27]. Furthermore, haplotype analysis of the inbred strains has shown the presence of over 60,000 haplotype blocks of varying size throughout the genome of inbred strains [28]. Since the presence of these blocks could be a source for overestimation of extent of genetic regulation and false positive associations, for each transcript and protein we removed significant associations due to high linkage disequilibrium (defined as R-squared of 0.5 or larger between genotypes). Since the transcript and protein data have different variance properties, which may subsequently affect our statistical power to detect associations in the two different datasets, we avoided the use of the same statistical cutoff for each dataset. Instead, in order to achieve a comparable genome-wide cutoff across the two datasets, we made use of false discovery rate and compared the two association results by restricting the genome-wide mapping results of each dataset to a list of associations with a similar false discovery rates. The results are summarized in Table 2 and the eQTL profile for the combined set is depicted in Figure 6A. At the 5% genome-wide FDR cutoff (p-value<1.7e-05) we identified 14463 associations for the transcript data (referred to as “eQTL” for expression QTL). At this cutoff stringency, 63% of the transcripts (6299 out of 9896) mapped to at least one locus and roughly one third of the transcripts (3651 out of 9896) mapped to two or more loci (Table 2). In contrast, at the same 5% FDR (p-value<9.6e-06), we only found 1368 significant associations for the proteins (referred to as “pQTL” for protein QTL). The fraction of total proteins with significant association was 672 genes (43%) from which 339 mapped to more than one locus (Table 2). In general, the mapping data for molecular phenotypes can be subdivided into “local eQTLs/pQTLs” to highlight the presence of genetic variation near/within the gene controlling the transcript or protein levels and “distant eQTLs/pQTLs” to discover trans-acting gene-locus interactions at the genetic level [29]. An empirical calculation of haplotype blocks in the HMDP panel (based on continuous stretch of SNPs with the R-squared value above 0.5) showed an average size of 0.73 Mb and a range from less than a kb to 11 Mb (median = 0.25 Mb). Given this fine mosaic structure in the HMDP genotypes, we defined a local eQTL/pQTL as an eQTL/pQTL with the peak SNP located in the 4 Mb window flanking 2 MB on either side of the transcription start site and transcription termination site of the gene. Based on this, from the total of 14463 significant associations in the transcript data, 2066 were local and 12397 were distant eQTLs. In the protein data the numbers of local and distant eQTLs were 144 and 1224, respectively. The proportion of variance explained by the peak SNP in local pQTLs was 44%, local eQTL was 42%, distant pQTL was 27%, and distant eQTL was 23%. The difference in proportion of variance explained between the distant pQTLs and distant eQTLs was highly significant (Student t-test p-value<1e-16), however, a similar comparison showed no significant difference between the mean effect sizes of local pQTL and local eQTLs (Figure S5). For each dataset, the proportion of variance explained by the local SNPs was significantly larger, as expected, as compared to the distant SNPs.

**Table 2:** Genome-wide association profiles for the proteome and the transcriptome data.

Global Analysis	Number of Probesets/Peptides	Total Number of Significant Associations	Number of Probesets/Peptides With at least One Significant Association (% Total Phenotypes)	Number of Probesets/Peptides with Local Associations (% Total Phenotypes)	Number of Distant Associations	Number of Probesets/Peptides With More Than One Significant Association (% Total Phenotypes)
Transcriptome	9896	14463	6299 (63%)	2066 (21%)	12397	3651 (37%)
Proteome	1543	1368	672 (43%)	144 (9%)	1224	339 (21%)

Transcript-Protein Pairs	Number of Genes (Number of Probesets/Peptides)	Total Number of Significant Associations	Number of Genes With at least One Significant Association (% Total Genes)	Number of Genes with Local Associations (% of Total Genes)	Number of Distant Associations	Number of Genes With More Than One Significant Association (% Total Phenotypes)
Transcriptome	396 (607)	878	297 (75%)	79 (20%)	799	205 (51%)
Proteome	396 (1343)	920	242 (61%)	46 (9%)	874	171 (43%)

### Absence of hotspot loci regulating numerous transcripts/proteins

A previous study in plants found that a small number of loci regulated the levels of many proteins [11]. Accordingly, we examined our data for the existence of similar “hotspots”. In the transcript data, we found that the 14463 eQTLs mapped to 9108 distinct peak SNPs. Over 85% of these SNPs (8034 out of 9108) were associated with either one or two transcripts and only a small fraction (334 SNPs) were associated with five or more transcripts. In the protein data, 1368 significant pQTLs mapped to 1088 distinct SNPs across the genome. From these 1088 SNPs, 930 were associated with a single protein, 100 were associated with 2 proteins, and 14 SNPs were associated with 5 or more proteins. From the 1368 peak markers associated with protein levels 438 (32%) were also a peak SNP for one or more transcripts. To investigate if the distinct peak SNPs found in the transcript and protein data map near each other, we divided the genome into 2 Mb bins and using a 50 kb sliding window counted the number of associations in each bin. In the transcript data, the median eQTL number/window was 8 and the highest number of associations was found for bins on Chr 4 (from 98.7 Mb to 100.8 Mb) with 71 eQTLs, Chr 5 (from 80 Mb to 83.1 Mb, from 112.4 Mb to 114.4 Mb) with 79 and 75 eQTLs in each respectively, Chr 7 (from 143.2 Mb to 146.2 Mb) with 78 eQTLs, Chr 8 (from 93.0 Mb to 95.1 Mb) with 71 eQTLs, Chr 17 (from 43.8 Mb to 46.4 Mb) with 80 eQTLs, and Chr 18 (from 55.0 Mb to 57.5 Mb) with 76 eQTLs. In the protein data, however, most associations were randomly distributed except for a clustering of associations on Chr 3 (from 36.5 Mb to 38.6 Mb) with 20 pQTLs and Chr 11 (from 94.3 Mb to 96.7 Mb, and from 114.1 Mb to 118.1 Mb) with 19 and 21 pQTLs respectively (Figure 6B). These results contrast to the previously published reports where hotspots containing hundreds or thousands of eQTLs were observed [30], [31]. This could be partially explained by both our ability to map molecular phenotypes with higher precision in the HMDP panel and the relatively stringent genome-wide threshold chosen to carry out the analysis. The eQTL hotspots on Chr 4 (from 98.7 Mb to 100.8 Mb) resides 6 Mb proximal and the Chr 5 hotspot (from 80 Mb to 83.1 Mb) resides 25 Mb distal to the Chr 5 hotspot reported recently in mouse-hamster radiation hybrid cell line [32]. Despite the relative close distance in mapping, however, we did not find a significant overlap between the genes mapping to these two loci in the two studies.

### Genetic regulation of transcript and protein levels

The global look at the eQTL profiles of the transcriptome and proteome described above suggested that transcripts are more extensively regulated at the genetic level than proteins. However, since the transcriptome data is more comprehensive than the protein data, the differences observed between two datasets might be due to sampling bias. In order provide a measure of similarity for genetic regulation of proteins and transcripts we restricted the data to the set of 396 genes for which we had both protein and transcript measurements available. As mentioned earlier, in this restricted dataset the 396 genes are represented by twice as many peptides as probesets (1343 peptides and 607 probesets). Similar to the genome-wide global analysis, we avoided the use of single statistical cutoff to compare association results across the transcript and peptide datasets, as each dataset has its own variance properties. Instead, we compiled separate lists of significant associations for each dataset using the same FDR cutoff. Since the FDR threshold is driven by the distribution of p-values in each dataset, this allowed us to compare the two lists directly without setting a single statistical cutoff for both datasets. Limiting the mapping data to those associations that met the 5%FDR cutoff in each dataset (p-value<1.7e-05 for transcripts and p-value<9.6e-06 for proteins) we found that despite mapping twice as many peptides as probesets the number of significant associations were roughly equal (939 and 1083 significant associations for probesets and peptides, respectively). This suggests that transcripts are twice as likely to be genetically regulated as are peptides. Next, we performed a gene level analysis where we assigned the associations obtained in each data set to their respective genes and for each gene investigated the degree of similarity in genetic regulation across the protein and transcript dataset. As summarized in Table 2 and consistent with the probeset/peptide analysis described earlier, we found that the number of genes under genetic regulation, as judged by fraction of total genes with at least one significant genome-wide association, favors the transcript dataset. Overall, from the initial 396 genes, 75% (297/396) of the genes with transcript products had at least one significant result vs 61% (242/396) of the genes in the LC-MS dataset. The number of genes with multiple eQTL and pQTL was 205 and 171, respectively. We also looked at the comparison across datasets after classifying the mapping results into local and distant eQTL and pQTL. For distant associations, 281 genes mapped to 799 distinct loci in the transcript dataset and 236 genes mapped to 874 unique genomic locations in protein dataset. Overlapping the association results from the two datasets for distant eQTL/pQTLs, we found that only 25 loci overlap with each other. From these 25 loci, 7 loci had the same peak SNP between the pQTL and the eQTL and in the remaining 18 the distance of peak SNP between the eQTL and pQTL ranged from 2.6 kb to 1.6 Mb. For local eQTLs, we found approximately twice as many local eQTLs as local pQTLs for the 396 genes (79 vs 46). To examine the extent of overlap between local QTLs, we considered a pQTL and an eQTL shared if they mapped within 2 Mb of each other. Using this definition, there were 26 local QTLs shared between the protein and transcript products of the gene. From these common QTLs, 8 mapped to the same peak SNP in the genome-wide association and 18 others mapped in various proximities of each other ranging from 23 bp to 1.8 Mb. The number of shared local QTLs suggests that majority of local pQTLs (26/46 = 56%) are likely to be conserved at the transcript level, and only 1/3 (26/79 = 32%) of eQTLs are conserved at the protein level.

Since local eQTLs are less likely to contain false positives [33], we utilized them to assess if our definition of significance based on FDR had any effect of the results of the comparative analysis we described above. For this, since the transcript local eQTL counts outnumbered the peptide counts, we set a fixed threshold for significance in the transcript dataset (5% FDR, p-value<1.69e-05), counted the number of pQTL overlaps with the significant eQTLs at varying statistical cutoffs, and asked if the increase in the overlap was more than what would be expected by chance. For this we examined results at 5% FDR cutoff, 10% FDR cutoff, and 25% FDR cutoff. At the 5%FDR (p-value<9.58e-06) there were 46 local pQTLs from which 26 overlapped with the 79 eQTLs. Decreasing the p-value stringency to detect association in the protein data, however, did not significantly increase the overlap between eQTL and pQTL. At the 10% FDR (p-value<2.95e-05) we detected 56 pQTL from which 28 overlapped with eQTLs (2 more than 5%FDR), and at the 25% FDR (p-value<0.0002) we detected 68 pQTLs for which the overlap with eQTL was only 29, one more than 10% FDR cutoff and 3 more than the 5%FDR cutoff. These non-significant changes in overlap between eQTL and pQTL suggest that the lack of overlap between eQTL and pQTL as presented earlier was not due to the genomewide significance thresholds set for each dataset.

We should emphasize that one limitation of our study originated from our study design where we utilized different number of mice per strain to estimate the transcript and the peptide levels. For transcript levels we profiled the RNA from 3 mice per strain and estimated transcript levels for genes by averaging over the data obtained for three mice, but for the LC-MS data we only sampled one mouse per strain. This design, by its nature, results in a higher power to detect genome-wide associations and significant correlations with clinical traits for transcript data in comparison to the peptide data. In fact, mapping transcript levels by taking only the data from one of the three microarray data for each strain gave us on average 36% fewer local eQTLs in comparison to what we had obtained by averaging the expression phenotypes over the three microarrays (Text S1 and Table S6). This difference would not change the overall conclusions regarding the commonality and the differences observed between the peptide and transcript genome-wide mapping results.

## Discussion

We report a comparative analysis of the genetic regulation of the transcriptome and proteome in a mammalian system. By examining the effects of thousands of genetic perturbations simultaneously on transcript and protein levels in the HMDP, we were able to investigate the global nature of relationships between the two. Since the HMDP was typed for numerous clinical/physiologic traits, we were also able to study the relationships of these to transcript levels as compared to protein levels. Finally, we examined the commonality of genetic drivers affecting transcript and protein levels. We discuss these points in turn below.

We performed the comparison of protein and transcript levels using two separate approaches. In one approach we comprehensively compared the LC-MS peptide measurements to the microarray expression estimates. In the second approach we examined the relationship between the expression of exons representing the peptides identified by the LC-MS to the expression of exons counted in the next generation sequence data. In addition, to address sources of technical and biologic variation in our measurements, we filtered peptides with significant nongenetic variation. In all these analyses we found that the relationship between the protein expression and transcript expression was modest at best and in only 50% of the cases did this relationship reach nominal statistical significance. We also found that the amount of genetic variation is a predictor of concordance between peptides and transcripts.

Our data complements the data previously published for yeast and plant indicating similar modest protein-transcript relationship. As compared to yeast, we found a slightly higher estimate of protein-transcript concordance (0.27 vs 0.18 correlation) when considering all the peptide measurements and significantly higher estimates (0.42 vs 0.18 correlation) when considering peptides with large genetic variability. The higher estimates reported here are likely to be more reflective of the true relationship between protein and transcript levels as compared to the previous reports mainly due to the choice of technology used to measure protein levels in our study. We utilized the differential labeling technique as put forth by Qian and colleagues where the label free samples are combined with an internal control labeled with heavy isotope [15]. This mixture is then quantified and the results are reported as the ratio of sample to the pool for each identified peptide during mass spectrometry. This strategy, which offers the advantage of overcoming peptide level variation due to platform robustness, has been shown to more precisely quantify peptides as compared to label free methods [15]. This was evident in our study as well where we showed that in general the variance in technical replicates was low, with an overall narrow distribution across the peptides quantified.

Biologically, the modest relationship between the proteins and transcripts is likely to be explained in part by molecular events such as translational efficiency, alternative splicing, folding, assembly into complexes, transport and localization, covalent modification, secretion, and degradation, all of which affect protein levels independently of transcripts. The importance of these post-transcriptional processes is highlighted by a recent report showing that the presence of genetic variation in some of these post-transcriptional processes is associated with certain human diseases [34]. We acknowledge that the design of our study and our most comprehensive dataset, which was generated by Affymetric microarrays capturing the 3′ end of transcripts, prevented us from comprehensively addressing the issue of differential splicing. However, using two complementary approaches, NGS and concordance level of peptides, we examined the possibility of differentially regulated isoforms as a predictor for the lack of concordance between microarray data and LC-MS data. In neither case did differential splicing appear to contribute importantly to the lack of transcript-protein correlation.

An unexpected finding was the stronger association of transcript levels with clinical traits as compared to protein levels with clinical traits. This is likely due in part to the greater technical difficulties for the quantification of proteins as compared to transcripts, but the differences were quite striking and there may be additional explanations. One possible explanation is that the molecular phenotypes are reactive to the clinical phenotypes (rather than being causal) and that there is increased buffering at the protein level. Apart from the strength of the trait associations, the protein and transcript associations in many cases did not overlap. For example, less than 15% of clinical trait-transcript correlations were replicated when traits were correlated with the corresponding proteins.

At the genetic level we also found marked differences in the number and locations of loci controlling protein and transcript levels. When we directly examined protein-transcript pairs corresponding to the same gene, we found that the transcript data had twice as many associations as the protein data. One plausible explanation for the existence of the differential genetic regulation between proteins and transcripts is that of “phenotypic buffering” as put forth previously [11]. An alternative explanation, however, would be that in general the more removed a phenotype is from the DNA variation, the more complex the phenotype becomes. Thus, protein levels would be affected by all the factors influencing transcript levels as well as numerous additional factors. The consequence of increasing complexity in the phenotype is that less of the variation in phenotype would become linked to a single DNA variation.

In summary, we highlighted the similarities and differences in genetic regulation of protein and transcript levels. Although a component of the observed differences in regulation is likely to be technical, particularly with respect to the protein levels, it is clear that the proteomics and transcriptomics provide nonoverlapping information. Thus, these data have important implications for systems biology approaches that utilize such high throughput data. They also raise fundamental questions about the complexity of the relationships between various biological scales involved in complex genetic traits.

## Materials and Methods

### Ethics statement

All animals were handled in strict accordance with good animal practice as defined by the relevant national and/or local animal welfare bodies, and all animal work was approved by the appropriate committee. All experiments in this paper were carried out with UCLA IACUC approval.

### Animals and clinical phenotype collection

Male mice from the HMDP panel, approximately 6–10 weeks of age, were purchased from Jackson Labs and were fed Purina Chow (Ralston-Purina Co., St. Louise, MO) at 16 weeks of age. All mice were maintained on a 12 h light/dark cycle. At 16 weeks of age, whole body fat, fluids and lean tissue mass of mice were determined using a Bruker Optics Minispec nuclear magnetic resonance (NMR) analyzer (The Woodlands, TX, USA) according to the manufacturer's recommendations. We also calculated the total mass of the mice, sum of lean mass, free fluid, and fat mass, and body fat percentage, fat mass/total mass. Following a 16-hour fast, mice were weighed and then bled retro-orbitally under isoflurane anaesthesia. Complete blood counts were performed using a Heska CBC-Diff analyzer (Heska Corp, Loveland, CO, USA). An external control sample with known analyte concentration was run in each plate to ensure accuracy. Glucose levels were determined using commercially available kits from Sigma (St Louis, MO, USA). Insulin levels were measured using commercial ELISA kits (ALPCO Diagnostics). All measurements were performed in triplicate according to the manufacturer's instructions. Plasma lipids were determined as previously described [35]. Mice were euthanized by cervical dislocation and the mass of individual tissues and fat depots (heart, kidney, retroperitoneal fat pad, epididymal fat pad, subcutaneous fat pad, and omental fat pad) were determined by dissecting and weighing each tissue/pad separately after the mice were euthanized. Following this, liver tissues were dissected out, flash frozen in liquid nitrogen, and kept at −70 degrees until further processing.

### RNA isolation, expression profiling, and RNA-Seq experiment

At 16 weeks of age, the liver tissues of the mice were dissected out, flash frozen in liquid nitrogen, and kept at −70 degrees until further processing. For RNA profiling the RNA from 3 mice per strain were hybridized to Affymetrix Mouse Genome HT_MG-430A arrays. Frozen liver samples were weighed and homogenized in Qiazol according to the manufacturer's protocol. Following homogenization, RNA extraction was performed using Qiagen's RNeasy kit (cat# 74104). Ninety two strains of mice had three biological replicates, five strains had two biological replicates and two strains had one biological replicate each. All RNA samples were cleaned using a Biosprint96 (Qiagen, Valencia, CA) with RNA cleanup beads (Agencourt Bioscience, Beverly, MA) following manufacturer's protocol with adaptations for use with the Biosprint. The quality of the total RNA from the samples was monitored by the Agilent 2100 Bioanalyzer (Agilent Technologies, Palo Alto, CA) and RNA quantity was measured with a NanoDrop (NanoDrop Technologies, Inc. Wilmington, DE) following the manufacturer's instructions. All samples were arrayed into three 96 well microtiter plates following a randomized design format that places samples from the same strain on different plates to better estimate variance across testing strains. All target labeling reagents were purchased from Affymetrix (Santa Clara, CA). Double-stranded cDNAs were synthesized from 1 ug total RNA through reverse transcription with an oligo-dT primer containing the T7 RNA polymerase promoter and double strand conversion using the cDNA Synthesis System. Biotin-labeled cRNA was generated from the cDNA and used to probe Affymetrix Mouse Genome HT_MG-430A arrays. The HT_MG-430A Array plate consists of 96 single MG-430A arrays arranged into standard SBS 96 well plate format. All cDNA and cRNA target preparation steps were processed on a Caliper GeneChip Array Station from Affymetrix. Array hybridization, washing and scanning were performed according to the manufacturer's recommendations. Scanned images were subjected to visual inspection and a chip quality report was generated by the Affymetrix's GeneChipOperating System (GCOS) and Expression console Affymetrix). Two of 288 chips were excluded due to low QC scores. The image data was processed using the Affymetrix GCOS algorithm utilizing quantile normalization or the Robust Multiarray method (RMA) to determine the specific hybridizing signal for each gene. Expression data can be obtained from Geo database (GSE16780). To avoid the effect of SNP on hybridization, we matched the location of ∼14 million SNPs from dbSNP database (NCBI) to the location of the individual probes on the genome. If the location of the probe had a matching SNP within it we flagged the probe and exclude it from the cdf file prior to RMA normalization. If a probeset contained SNP in 8 or more 25-mer probes, we excluded the probeset from the analysis. The cleaned datasets were then background corrected and normalized using the affy package (from bioconductor) using rma, pmonly, and median-polish normalization methods.

RNA isolation for Next Generation Sequencing followed the same protocol as the one described above for the microarray data. For the RNA-Seq experiment, two inbred mice (C57BL/6J and DBA/2J) were chosen. Library preparation was carried using illumina's mRNA-Seq 8-Sample Prep Kit protocol (Illumina, cat# RS-100-0801). In brief, 1 to 10 ug of RNA was used for library construction. In the first step the poly-A containing mRNA molecules were purified using poly-T oligo-attached magnetic beads. Next, the purified mRNA was fragmented into small pieces using divalent cations, followed by double stranded cDNA synthesis using random primers, adenylated at the 3′ end and ligated to the sequencing adapters. The ligated products were then separated on 2% agarose gel, 200 bp fragments were selected and PCR amplified using PE 1.0 and PE 2.0, and purified using QIAquick PCR Purification Kit (QIAGEN, part # 28104). The final library concentration was verified by Bioanalyzer. Sequencing reaction was performed by Illumina Genome Analyzer 2.0 at UCLA Human Genetics microarray core. Raw sequences were uploaded onto Galaxy website (at http://main.g2.bx.psu.edu/) and using the Tophat software [22] was aligned against the reference genome (*M. musculus*, *mm9*) downloaded from UCSC. Alignment was performed by setting the parameter for misalignment to one. Relative abundance of transcripts (in Fragments Per Kilobase of Exon per Million read sequence units) was estimated using the Cufflink software [23] and the Ensembl's Mus_musculus NCBIM37 as the reference annotation file.

### Protein isolation and sample preparation

Male mice were euthanized using isoflurane followed by cervical dislocation at 6–10 weeks of age. The liver tissue was immediately frozen in dry ice until further processing. The 97 samples corresponding to different mouse strains plus some extra samples from C57BL/6J mouse were randomized into 10 batches of 10 samples. Each batch was processed separately prior to quantitative LC-MS analysis. Before the LC-MS analysis the batches were put together and the sample list was randomized one more time. The extraction and digestion of the proteins was performed using a commonly used protocol based on denaturation of protein in 8 M urea followed by digestion with trypsin. Briefly, approximately 5 mg of liver tissue was resuspended in 100 ul denaturing solution (8 M urea, 50 mM Tris-HCl pH 8.0 and 1 mM EDTA) and homogenized with a motorized pestle. Upon homogenization, the total protein content was measured by Bicinchoninic Protein Assay (BCA, Pierce, Rockford, IL) and the 500 ug aliquots were taken from each sample for further processing. DTT was added to a concentration of 10 mM in sample, then to solubilize and unfold the proteins the samples were incubated for 30 min at 37oC with shaking. Cysteine residues were alkylated by adding iodoacetamide up to 40 mM concentration and incubating for 1 hour at 37oC, with shaking, in the dark. For protein digestion the samples were diluted 10-fold with 50 mM ammonium bicarbonate (pH 7.8), supplemented with 1 mM CaCl2, 10 ug of trypsin and incubated for 3 hours at 37oC with shaking. The sample digests were purified with solid phase extraction using C18 columns (Discovery DSC-18, SUPELCO, 52601-U), lyophilized and resuspended in 25 mM ammonium bicarbonate pH 7.8. The peptide amounts were estimated with BCA assay. On the average the amount of purified tryptic peptides was 200 ug. To generate the 18O reference sample, 20 ug of each sample was pooled, then boiled 10 minutes, followed by immediate cooling for 10 minutes. The boiling/cooling steps were performed to inactivate trypsin (this step helps to avoid back-exchange of 18O-labeled peptides). The pooled reference was then subjected to solution-phase tryptic 18O exchange, followed by quenching of tryptic activity with formic acid. The pooled sample was then added in equal amounts with each individual sample for quantitation purposes.

### Characterization of the mouse liver proteome

Construction of a library of proteins and tryptic peptides present in the liver is an important step for follow-up quantitation. 10 ug aliquots from all 97 strains were pooled together and subjected to LC fractionation by strong cation exchange (SCX) chromatography on a 200 mm×2.1 mm Polysulfoethyl A column (PolyLC, Columbia, MD) preceded by a 10 mm×2.1 mm guard column, using a flow rate of 0.2 mL/min. LC separations were performed using an Agilent 1100 series HPLC system (Agilent, Palo Alto, CA). Mobile phase solvents consisted of (A) 10 mM ammonium formate, 25% acetonitrile, pH 3.0 and (B) 500 mM ammonium formate, 25% acetonitrile, pH 6.8. Once loaded, isocratic conditions at 100% A were maintained for 10 min. Peptides were separated by using a gradient from 0–50% B over 40 min, followed by a gradient of 50–100% B over 10 min. The gradient was then held at 100% solvent B for another 10 min. Following lyophilization, all thirty fractions collected during this gradient were dissolved in 25 mM ammonium bicarbonate and stored at −80OC.

Each SCX fraction was analyzed with an automated custom-built capillary HPLC system coupled online to an LTQ ion trap mass spectrometer (Thermo Fisher, San Jose, CA) by using an electrospray ionization interface. The reversed phase capillary column was prepared by slurry packing 3-µm Jupiter C18 particles (Phenomenex, Torrance, CA) into a 75 µm i.d.×65 cm fused silica capillary (Polymicro Technologies, Phoenix, AZ). The mobile phase solvents consisted of (A) 0.2% acetic acid and 0.05% TFA in water and (B) 0.1% TFA in 90% acetonitrile. An exponential gradient was used for the separation, which started with 100% A, and gradually increased to 60% B over 100 min. The instrument was operated in a data-dependent mode with an m/z range of 400–2000. Ten most abundant ions from each MS scan were selected for further MS/MS analysis by using a normalized collision energy setting of 35%. Dynamic exclusion was applied to avoid repeat analyses of the same abundant precursor ion.

The SEQUEST software (Thermo Fisher) was used to search the MS/MS data against the mouse International Protein Index (IPI) database (version 3.52 http://www.ebi.ac.uk/IPI). Human keratins and porcine trypsin were added into the database as expected contaminants. Trypsin cleavage specificity was required for all of the considered peptides. The following criteria were used to filter raw SEQUEST results: 1) Xcorr≥1.9 and DeltaCn2≥0.21 for charge state +1; 2) Xcorr≥2.5 and DeltaCn2≥0.26 for charge state +2; 3) Xcorr≥2.8 and DeltaCn2≥0.32 for charge state +3. These criteria provide the maximum number of peptide identifications not exceeding 1% false discovery rate (FDR). To estimate the FDR of peptide identifications we searched against a reversed database as previously described [36].

### Relative protein abundance quantitation

Relative peptide and protein quantitation was based on ratios between intensities of natural ^16^O isotope containing peptides and reference peptides labeled with stable ^18^O isotope at the carbonyl group at the C-terminus of the peptide. To create a reference sample we pooled together 20 ug aliquots from all strains and labeled the C-termini with ^18^O isotopes using trypsin catalyzed exchange in the presence of heavy H_2_^18^O water as described above and elsewhere [37]. Prior to the LC-MS analysis 3.75 ug aliquots from each individual sample were mixed with the same amount of 18O-labeled reference sample.

The 7 ug aliquots were analyzed on a LTQ-Orbitrap mass spectrometer that was interfaced with a 75 um i.d.×65 cm long LC column packed with 3 um Jupiter C18 particles (Phenomenex). The mobile phase solvents consisted of (A) 0.2% acetic acid and 0.05% TFA in water and (B) 0.1% TFA in 90% acetonitrile. An exponential gradient was used for the separation, which started with 100% A and gradually increased to 60% B over 100 min. LC-MS datasets were analyzed by in-house software VIPER [38] that detected features in mass – elution time space and assigned them to peptides in AMT tag database as described elsewhere [39], [40]. Typically an LC-MS run identifies ∼3,500 16O/18O peptide pairs that co-elute with a 4.0085 Da mass difference.

As we mentioned before, the relative abundances of tryptic peptides were calculated as the ratio between light and heavy isotopes. The relative abundances then were normalized with EigenMS procedure [41] to correct systematic biases that may arise for example from unequal sample loading, batch-to-batch differences in sample processing and LC column variability. Briefly, the EigenMS procedure discovers the systematic trends (so-called eigenpeptides) in the data using singular value decomposition and then removes contributions of those eigenpepides from each peptide. For all data analysis purposes the peptide and protein intensities were log2 transformed and zero-centered by subtracting the peptide or protein specific means taken across all the samples.

### Immunobloting experiments

To determine the protein levels by immunobloting, liver samples were homogenized in RIPA including phosphatase and protease inhibitors (Santa Cruz Biotech sc-24948), and protein determination were done using the Biorad Dc Assay. Protein samples were boiled following addition of Laemmli loading dye, separated on Invitrogen precast gels, and transferred to PVDF membranes. Membranes were rinsed in 1× TBST (Cell signaling #9997) blocked in 5% skim milk-TBST, rinsed in TBST, and incubated with primary antibodies diluted in 3% BSA-TBST for 1 hr at 23degC or overnight at 4degC. Membranes were washed in TBST and incubated with an HRP-conjugated anti rabbit IgG KPL (#474-1516) 1/5000 in 5% skim milk-TBST. Membranes were washed again, incubated in ECL-plus, and signal detected using a Biorad Chemidoc or film. Densitometry was done using the Biorad Quantity One software. The following list of antibodies and working dilutions were used for each protein: Fasn (Cell Signaling cat #3180, 1/2000), Acyl (Cell Signaling cat #4332, 1/2000), Ywhae (Cell Signaling cat #9635, 1/2000), Vim (Cell Signaling cat#3932, 1/1000), Rkip (Cell Signaling cat#5291, 1/2000), Gapdh (Cell Signaling cat#3683, 1/5,000), Glo1 (Sigma Chemical SAB1100242, 1/20,000), GstA4 (Sigma Chemical SAB1100244, 1/20,000), AnxA5 (Sigma Chemical AV36687, 1/2000), Hao1 (Sigma Chemical AV42480, 1/2000), Aldh3A2 (Sigma Chemical HPA014769, 1/20,000), Actin (Sigma Chemical A2066, 1/5,000), Acox1 (Abnova PAB4367, 1/2000).

### Data filtering

For transcript data we applied three filtering steps based on 1) genetic heritability, 2) probeset annotation. We have profiled 3 mice per strain which allowed us to estimate the broad sense heritability for each transcript. Broad sense heritability for each transcript was measured using ANOVA where strain information was used as a grouping factor. The broad sense heritability which is defined as the ratio of genetic variance over total variance for the phenotype was estimated by dividing the sum of squares of the strain information factor over total sum of squares in the ANOVA. The significance of heritability was established if the p-value for the strain information term in ANOVA was below the nominal 0.05 threshold. The selection cutoff for including gene in the analysis based on heritability was set to heritability p-value of 0.05. From the 22700 probesets 10186 probesets did not meet this cutoff. For annotation filtering, we acquired the Ensembl Gene ID for each Affymetrix probeset and selected those probesets that were only annotated to only one Ensembl gene. From the initial 22700 probesets, 4401 probesets had ambiguous annotation (either did not map to a gene or mapped to more than 2 Ensembl genes). Overall, 9896 probesets met both filtering criteria (significant heritability and unique Ensembl annotation). For the protein data, the initial filtering steps were based on 1) eliminating peptides with excessive missing values which would otherwise have unreliable mapping information, 2) eliminating peptides with missed internal cleavage sites which cause unreliable measurement. To annotate peptides we utilized the SpliceCenter web-based tool [42] to obtain the location of the exon each peptide represents. Peptides which mapped to multiple exons of more than one gene (as determined by SpliceCenter) were excluded from the analysis because of ambiguous annotation. The genomic exon coordinates for each peptide was then used to query the Ensembl database to acquire the Ensembl Gene IDs. In the second step of filtering peptides that had more than the value of 2 for the ratio of total variance over technical variance were chosen. Overall, 1543 peptides met the two-stage filtering described above.

### Genotyping and genome-wide association mapping

Inbred strains were previously genotyped by the Broad Institute (http://www.broadinstitute.org/mouse/hapmap), and they were combined with the genotypes from Wellcome Trust Center for Human Genetics (WTCHG). Genotypes of RI strains at the Broad SNPs were inferred from WTCHG genotypes by interpolating alleles at polymorphic SNPs among parental strains, calling ambiguous genotypes missing. Of the 140,000 SNPs available, 95,854 were informative with an allele frequency greater than 10% and missing values in less than 10% of the strains. These SNPs were used for both protein and transcript genome-wide association analysis.

We applied the following linear mixed model to account for the population structure and genetic relatedness among strains in the genome-wide association mapping [27]: y = μ+xβ+u+e.

In the formula, μ represents mean, x represents SNP effect, u represents random effects due to genetic relatedness with Var(u) = σ_g_^2^K and Var(e) = σ_e_^2^, where K represents IBS (identity-by-state) matrix across all genotypes in the HMDP panel. A restricted maximum likelihood (REML) estimate of σ_g_^2^ and σ_e_^2^ are computed using EMMA, and the association mapping is performed based on the estimated variance component with a standard F-test to test β≠0. We applied EMMA (Efficient Mixed Model Association) as an R implementation of a linear mixed model. The percent of variance explained for each molecular phenotype was calculated using the SNP effect calculated from EMMA by defining it as 1-(variance of residuals/variance of original phenotypes). It should be noted that since EMMA is orders of magnitude faster than other implementations commonly used, we were able to perform statistical analyses for all pairs of transcripts and genome wide markers in a few hours using a cluster of 50 processors. Both pQTL and eQTL were defined as “local” if the peak association SNP position was within a 4 Mb interval, flanking 2 Mb on either side of the transcription start and end of the gene under regulation. *Genome-wide cutoff:* Genome-wide cutoffs were calculated as the false discovery rates using the “qvalue” package for FDR calculation in the R statistical software [43]. Due to the computational complexity associated with evaluating q-values for over 400 million p-values, we computed the FDRs by taking the average FDR for 100 samples each containing 5 million randomly selected p-values from the original calculated p-values. FDR calculation was carried out separately for the protein and transcript dataset.

### GO analysis and other statistical methods/software

All statistical analyses and data visualizations were carried out using the R statistical software (available at http://cran.r-project.org/). Classification of proteins and transcripts to various GO categories was accomplished using Mouse Genome Informatics website at Jackson Laboratories and the GO ontology tool at Princeton. For each probe and each peptide, we first obtained the MGI IDs using the MGI batch query tool at http://www.informatics.jax.org/ [44]. Using MGI IDs we utilized the GO Term Mapper at http://go.princeton.edu, which is based on map2slim algorithm [45] to obtain the GO annotations and summary statistics. The background geneset used in this analysis was the list of all genes annotated by MGI. To assess the significance of the correlation coefficients observed in the PT-pair GO analysis, for each GO category we created 100,000 bootstrap datasets each equal in size to the number of genes assigned to the GO term. Bootstrapping was carried out randomly and without replacement from the pool of 584 original correlation p-values among the PT-pairs. The significance of the observed average p-values for each GO term is reported as the two-tailed test against the empirical distribution created by the corresponding 100,000 permutation set. All the correlation coefficients and corresponding p-values reported in the paper are calculated using the bicor function in the WGCNA R package [46]. The main advantage of using bicor, which performs biweight midcorrelation calculation, over Pearson's correlation is based the robustness of the correlation coefficient measurement to the presence of outliers in the data.

## Supporting Information

Dataset S1 Peptide expression values as measured by the LCMS.
(TXT)

Dataset S2 Peptide annotations.
(TXT)

Dataset S3 SNP information for the Affymetrix probes.
(XLS)

Figure S1 Immunoblotting validation results.
(DOC)

Figure S2 Cellular compartment representation of the measured proteins and transcripts.
(TIF)

Figure S3 The highest correlation between protein and transcript levels.
(TIF)

Figure S4 Average correlations of the transcript and protein product of the genes grouped by assigned GO categories.
(TIF)

Figure S5 Distribution of the SNP effects for local and distant eQTLs and pQTLs.
(TIF)

Table S1 Concordance between immunoblot experiments and LCMS data.
(DOC)

Table S2 Immunoblotting results.
(DOC)

Table S3 Biological representation of protein and transcript datasets.
(DOC)

Table S4 Correlation analysis of the GO terms for the protein-transcript pairs.
(DOC)

Table S5 Effect of SNPs in Affymetrix probes on eQTL detection.
(DOC)

Table S6 Effect of biologic replicates on eQTL detection.
(DOC)

Text S1 Supplementary material—SNP effect analysis.
(DOC)
