# Supplementary material for: Comparative Analysis of Proteome and Transcriptome Variation in Mouse
Source: PLoS Genet. 2011 Jun 9;7(6):e1001393. doi: 10.1371/journal.pgen.1001393 (PMC3111477; doi:10.1371/journal.pgen.1001393)

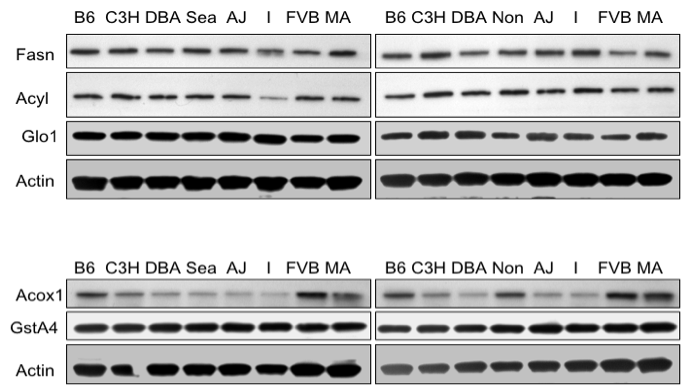
**Figure S1. Immunoblotting Experiments. The following results are the scanned picture of blots (2 replicates per protein) for various 11 proteins in 9 inbred strains. Quantification results are reported in TableS2.**


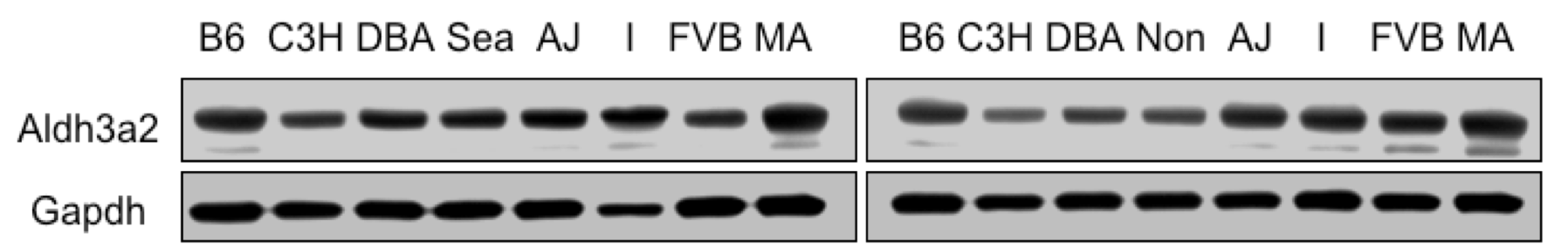

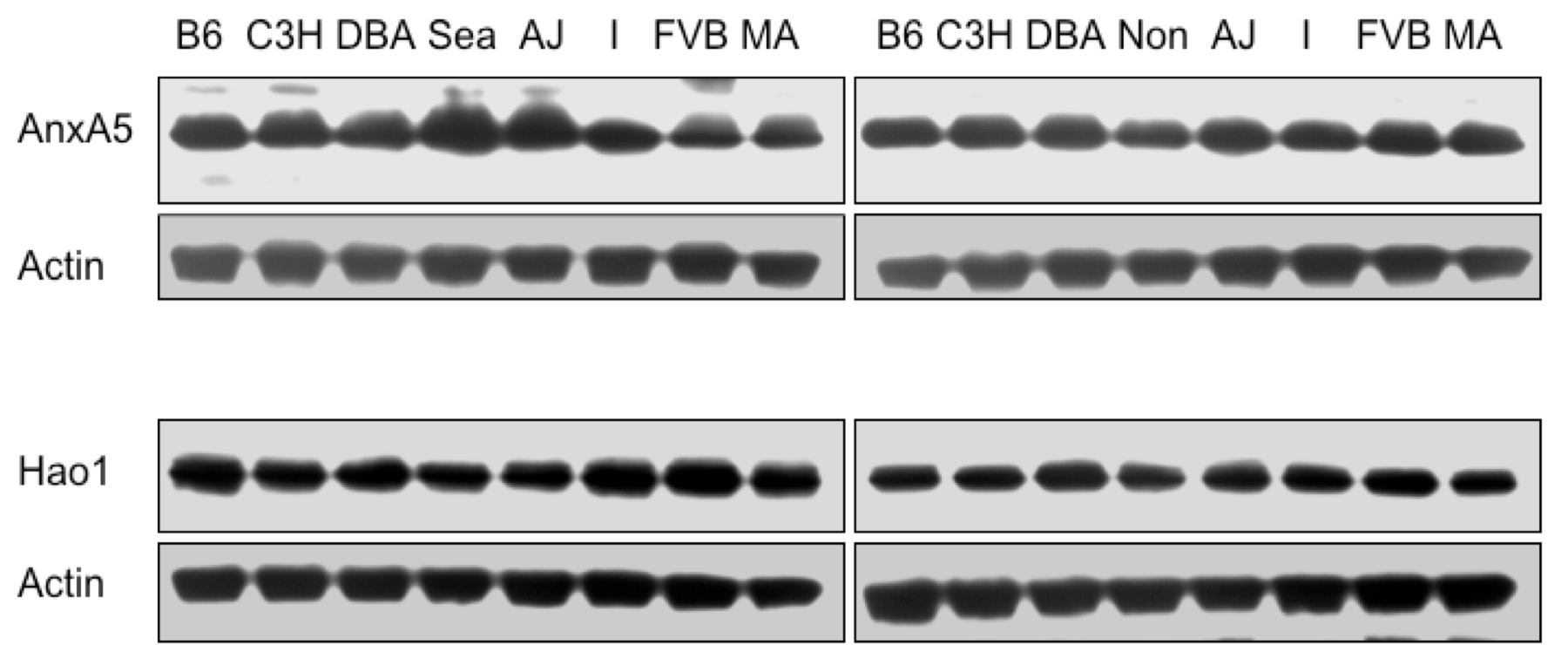


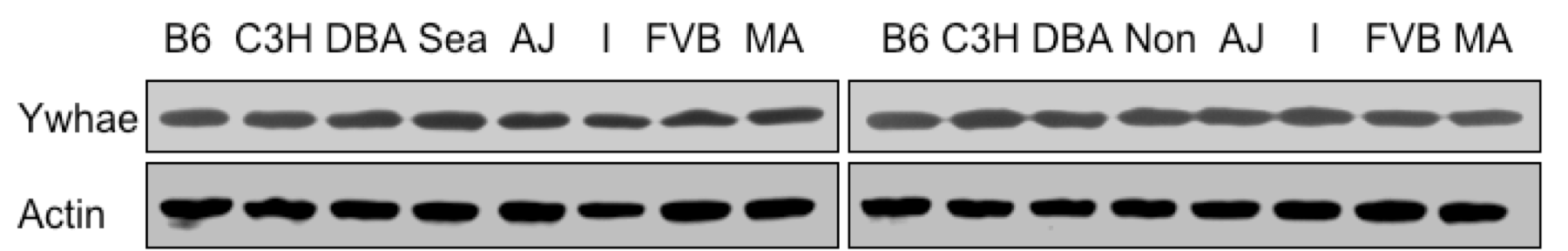


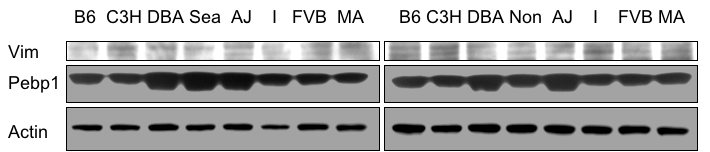

Supplement: Figure S1 — Immunoblotting validation results. (DOC) [file pgen.1001393.s004.doc]
