# Supplementary figures and images for: Comparative Analysis of Proteome and Transcriptome Variation in Mouse
Source: PLoS Genet. 2011 Jun 9;7(6):e1001393. doi: 10.1371/journal.pgen.1001393 (PMC3111477; doi:10.1371/journal.pgen.1001393)

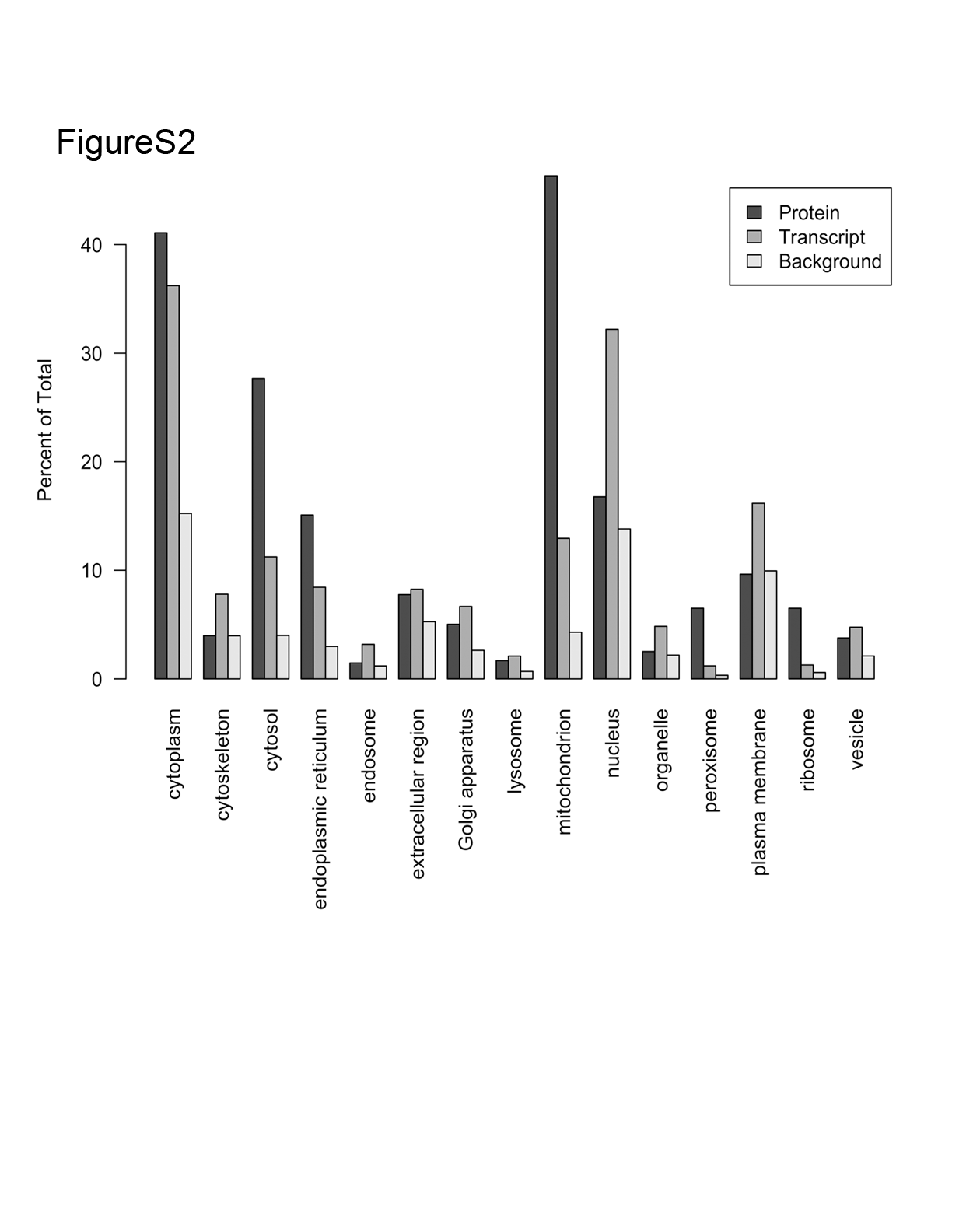

Supplement: Figure S2 — Cellular compartment representation of the measured proteins and transcripts. (TIF) [file pgen.1001393.s005.tif]

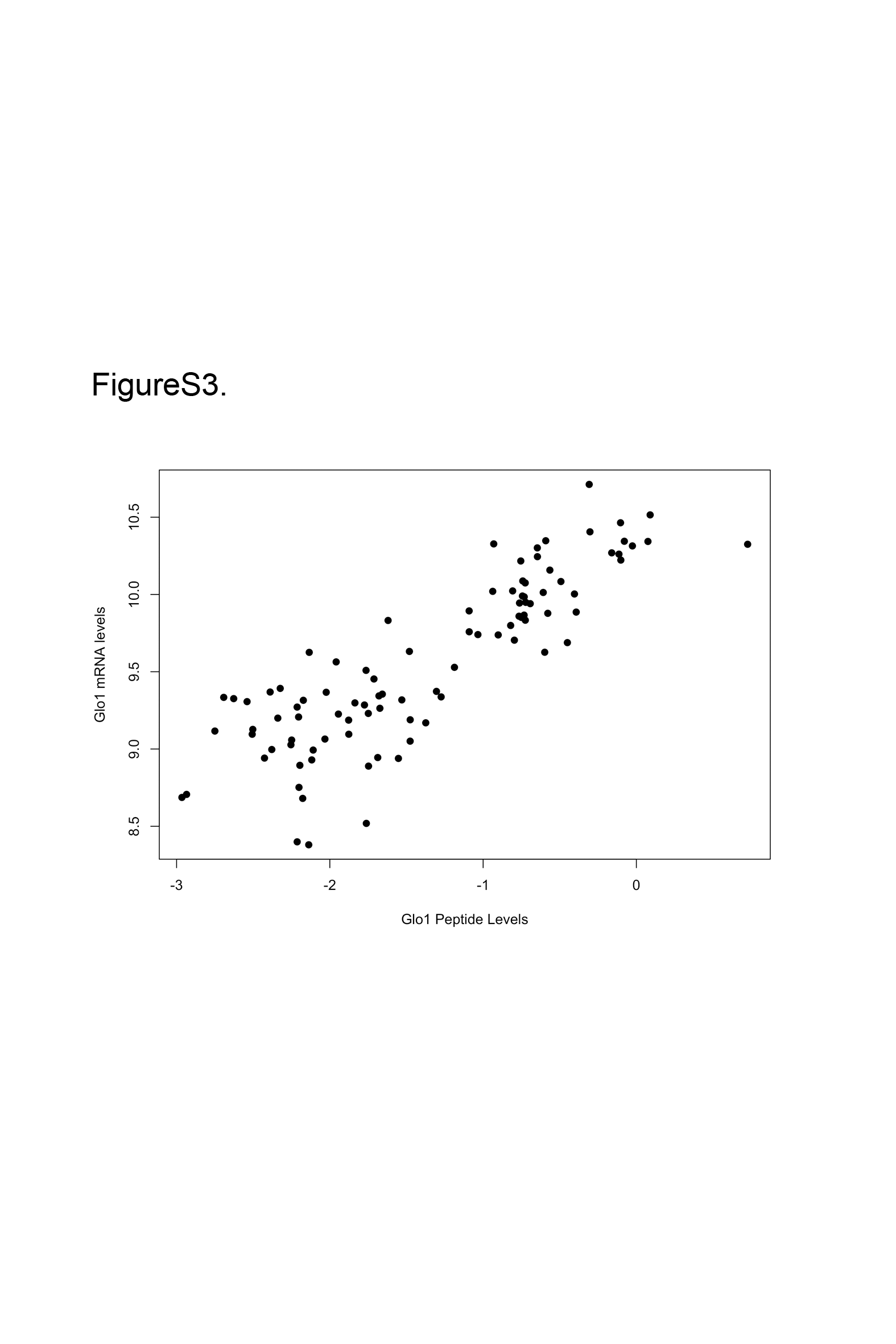

Supplement: Figure S3 — The highest correlation between protein and transcript levels. (TIF) [file pgen.1001393.s006.tif]

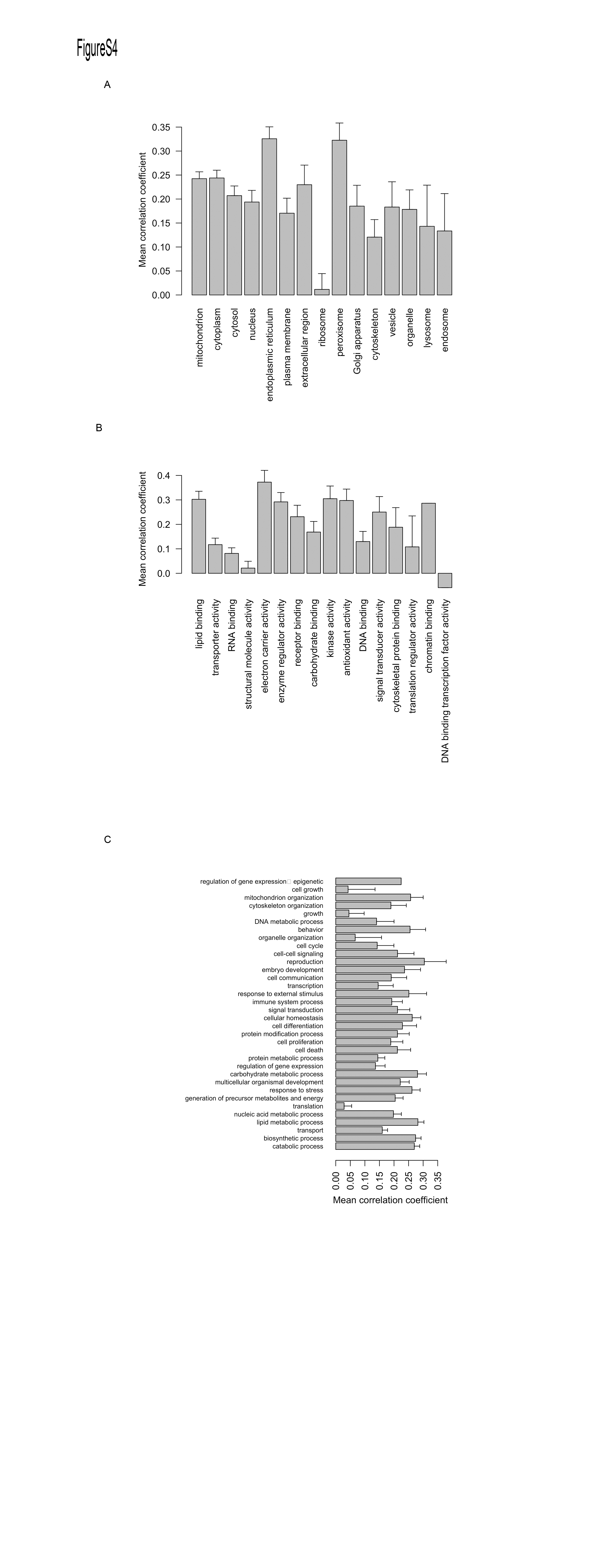

Supplement: Figure S4 — Average correlations of the transcript and protein product of the genes grouped by assigned GO categories. (TIF) [file pgen.1001393.s007.tif]

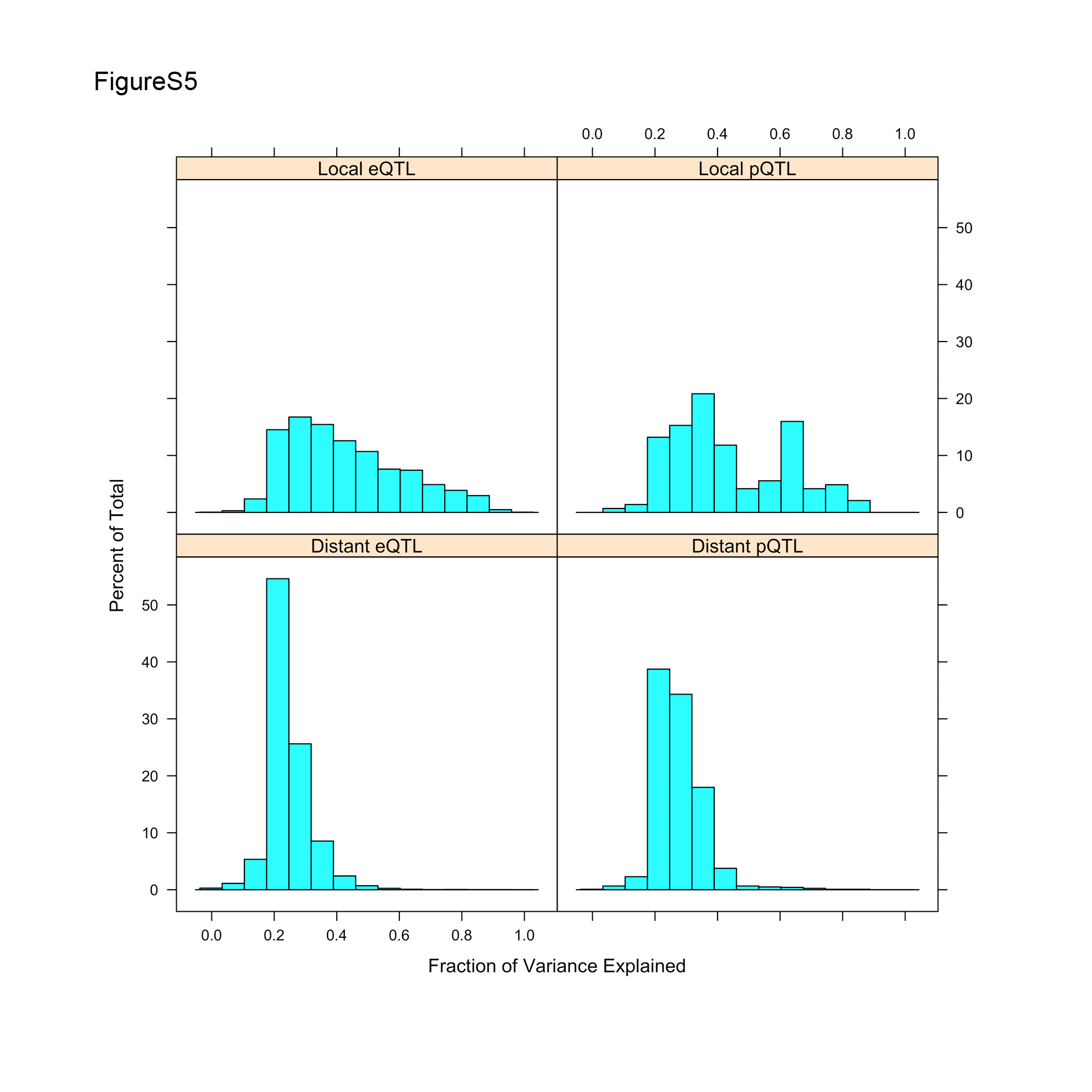

Supplement: Figure S5 — Distribution of the SNP effects for local and distant eQTLs and pQTLs. (TIF) [file pgen.1001393.s008.tif]
