# Supplementary material for: Comparative Analysis of Proteome and Transcriptome Variation in Mouse
Source: PLoS Genet. 2011 Jun 9;7(6):e1001393. doi: 10.1371/journal.pgen.1001393 (PMC3111477; doi:10.1371/journal.pgen.1001393)
Supplement: Table S1 — Concordance between immunoblot experiments and LCMS data. (DOC) [file pgen.1001393.s009.doc]

**Table S1. Correlation between LC-MS and Immunoblot results:**

| Western Data | MS_MS_Data | Spearman Correlation | Signal to Noise in All HMDP | Signal to Noise in 9 HMDP strains |
| --- | --- | --- | --- | --- |
| Aldh3a2-Western | Aldh3a2-EKEILAAIAADLSK | 0 | 3.97 | 0.28 |
| Aldh3a2-Western | Aldh3a2-EKPLALYVFSR | 0.33 | 7.72 | 0.47 |
| Aldh3a2-Western | Aldh3a2-HLTPVTLELGGK | -0.03 | 3.59 | 0.41 |
| Aldh3a2-Western | Aldh3a2-IVMEAAAK | -0.5 | 2.29 | 0.99 |
| Aldh3a2-Western | Aldh3a2-NVDEAINFINDR | -0.39 | 3.16 | 0.26 |
| Aldh3a2-Western | Aldh3a2-VMQEEIFGPILPIVSVK | 0.08 | 10.81 | 0.34 |
| Aldh3a2-Western | Aldh3a2-YLAPTILTDVDPNSK | -0.8 | 12.12 | 0.58 |
| Hao1-Western | Hao1-GVQDVLEILKEEFR | 0.26 | 6.15 | 1.51 |
| Anxa5-Western | Anxa5-ETSGNLEQLLLAVVK | -0.33 | 2.20 | 0.57 |
| Anxa5-Western | Anxa5-GLGTDEDSILNLLTSR | 0.24 | 12.13 | 0.74 |
| Anxa5-Western | Anxa5-GTVTDFPGFDGR | -0.1 | 4.26 | 0.37 |
| Anxa5-Western | Anxa5-SIPAYLAETLYYAMK | -0.26 | 1.94 | 0.28 |
| Anxa5-Western | Anxa5-VLTEIIASR | -0.14 | 2.90 | 0.75 |
| Vim-Western | Vim-ISLPLPTFSSLNLR | -0.29 | 1.74 | 0.56 |
| Pebp1-Western | Pebp1-FKVETFR | 0.68 | 9.77 | 0.72 |
| Pebp1-Western | Pebp1-GNDISSGTVLSDYVGSGPPSGTGLHR | 0.5 | 5.92 | 1.28 |
| Pebp1-Western | Pebp1-LYEQLSGK | 0.86 | 21.52 | 1.09 |
| Pebp1-Western | Pebp1-LYTLVLTDPDAPSR | 0.64 | 30.77 | 0.71 |
| Pebp1-Western | Pebp1-VDYAGVTVDELGK | 0.58 | 12.77 | 1.07 |
| Ywhae-Western | Ywhae-LICCDILDVLDK | -0.64 | 5.23 | 0.57 |
| Acly-Western | Acly-DLVSSLTSGLLTIGDR | 0.24 | 3.90 | 0.66 |
| Acly-Western | Acly-IGNTGGMLDNILASK | -0.12 | 4.20 | 0.66 |
| Acly-Western | Acly-LGLVGVNLSLDGVK | -0.14 | 0.73 | 0.60 |
| Acly-Western | Acly-SFDELGEIIQSVYEDLVAK | -0.09 | 2.44 | 1.27 |
| Acly-Western | Acly-TIAIIAEGIPEALTR | 0.25 | 2.34 | 0.58 |
| FASN-Western | Fasn-ACVDTALENLSTLK | 0.5 | 1.47 | 1.00 |
| FASN-Western | Fasn-AVAHILGIR | 0.83 | 5.16 | 0.81 |
| FASN-Western | Fasn-ELSFAAVSFYHK | 0.54 | 12.93 | 1.14 |
| FASN-Western | Fasn-FDASFFGVHPK | 0.25 | 2.62 | 1.29 |
| FASN-Western | Fasn-GVDLVLNSLAEEK | 0.68 | 2.46 | 0.99 |
| FASN-Western | Fasn-LGPVGGVFNLAMVLR | 0.54 | 12.06 | 0.91 |
| FASN-Western | Fasn-LLLPEDPLISGLLNSQALK | 0.43 | 7.15 | 0.83 |
| FASN-Western | Fasn-SDEAVKPLGVK | 0.94 | 8.66 | 1.35 |
| FASN-Western | Fasn-TGGLAFHSYFMEGIAPTLLQALK | 0.55 | 2.48 | 0.41 |
| FASN-Western | Fasn-TLEAVQDLLEQGR | 0.96 | 13.02 | 1.23 |
| FASN-Western | Fasn-TLLEGSGLESIINIIHSSLAEPR | 0.43 | 2.98 | 0.62 |
| FASN-Western | Fasn-VHLTGINVNPNALFPPVEFPAPR | 0.79 | 3.02 | 0.73 |
| FASN-Western | Fasn-VSVHIIEGDHR | 0.71 | 16.11 | 1.18 |
| FASN-Western | Fasn-YHGNVTLLR | 0.57 | 1.35 | 0.96 |
| GstA4-Western | Gsta4-DGHLLFGQVPLVEIDGMMLTQTR | -0.6 | 3.50 | 0.34 |
| GstA4-Western | Gsta4-KPPPDGPYVEVVR | 0.15 | 4.51 | 0.82 |
| GstA4-Western | Gsta4-YFPVFEK | 0.18 | 4.75 | 0.32 |
| GstA4-Western | Gsta4-YNLYGK | 0.1 | 6.85 | 0.13 |
| Acox1-Western | Acox1-AAATFNPELITHILDGSPENTR | 0.8 | 75.21 | 1.74 |
| Acox1-Western | Acox1-AAATFNPELITHILDGSPENTRR | -1 | 0.28 | 0.12 |
| Acox1-Western | Acox1-AFTTWTANAGIEECR | 0 | 2.88 | 0.49 |
| Acox1-Western | Acox1-ASEAHCHYVTVK | 0.71 | 7.19 | 1.09 |
| Acox1-Western | Acox1-DVTLGSVLGR | 0.9 | 34.76 | 0.60 |
| Acox1-Western | Acox1-EFGIADPEEIMWFK | 0.9 | 19.61 | 0.60 |
| Acox1-Western | Acox1-EIENLILNDPDFQHEDYNFLTR | 0.87 | 10.69 | 0.86 |
| Acox1-Western | Acox1-EVAWNLTSVDLVR | 0.76 | 17.65 | 0.83 |
| Acox1-Western | Acox1-FGYEEMDNGYLK | 0.82 | 10.81 | 0.80 |
| Acox1-Western | Acox1-GECYGLHAFVVPIR | 0.92 | 11.00 | 0.67 |
| Acox1-Western | Acox1-GHPEPLDLHLGMFLPTLLHQATEEQQER | 0.4 | 0.81 | 2.43 |
| Acox1-Western | Acox1-ILELLTVTRPNAVALVDAFDFK | 0.93 | 38.74 | 0.76 |
| Acox1-Western | Acox1-INESIGQGDLSELPELHALTAGLK | 0.93 | 9.91 | 1.01 |
| Acox1-Western | Acox1-IYDQVQSGK | 0.76 | 1.03 | 1.25 |
| Acox1-Western | Acox1-LTYGTMVFVR | 0.76 | 6.49 | 1.07 |
| Acox1-Western | Acox1-LVGGMVSYLNDLPSQR | 0.8 | 15.79 | 1.05 |
| Acox1-Western | Acox1-NLCLLYSLYGISQK | 0.86 | 9.17 | 0.63 |
| Acox1-Western | Acox1-NLQAQVSHR | 0.4 | 11.51 | 1.43 |
| Acox1-Western | Acox1-SFLVGSAAQSLSK | 0.75 | 4.74 | 0.36 |
| Acox1-Western | Acox1-TQEFILNSPTVTSIK | 0.95 | 43.53 | 0.65 |
| Acox1-Western | Acox1-YDGNVYENLFEWAK | 0.97 | 63.25 | 0.84 |
| Glo1-Western | Glo1-DFLLQQTMLR | 0.63 | 8.04 | 0.36 |
| Glo1-Western | Glo1-FSLYFLAYEDKNDIPK | 0.48 | 12.96 | 0.55 |
| Glo1-Western | Glo1-GFGHIGIAVPDVYSACK | 0.64 | 18.29 | 0.64 |
| Glo1-Western | Glo1-RFEELGVK | 0.64 | 24.89 | 0.58 |
| Glo1-Western | Glo1-TAWTFSR | 0.6 | 8.06 | 0.74 |
| Glo1-Western | Glo1-VLGLTLLQK | 0.62 | 39.87 | 0.49 |
