# Supplementary material for: Comparative Analysis of Proteome and Transcriptome Variation in Mouse
Source: PLoS Genet. 2011 Jun 9;7(6):e1001393. doi: 10.1371/journal.pgen.1001393 (PMC3111477; doi:10.1371/journal.pgen.1001393)
Supplement: Table S2 — Immunoblotting results. (DOC) [file pgen.1001393.s010.doc]

**TableS2. Quantification results (the values in the table are calculated after normalizing by the respective control housekeeping gene):**

| Strains | Glo1 | Acox1 | GstA4 | FASN | Acly | Ywhae | Pebp1 | Vim | Anxa5 | Hao1 | Aldh3a2 |
| --- | --- | --- | --- | --- | --- | --- | --- | --- | --- | --- | --- |
| B6 | 1.06 | 1.05 | 1.10 | 1.64 | 1.08 | 0.94 | 0.93 | 0.68 | 1.08 | 1.01 | 0.81 |
| C3H | 1.20 | 0.85 | 1.38 | 2.23 | 1.44 | 1.16 | 1.05 | 1.13 | 1.00 | 1.39 | 0.50 |
| DBA2J | 1.17 | 0.49 | 1.25 | 1.40 | 1.16 | 0.93 | 1.11 | 0.65 | 1.00 | 1.23 | 0.64 |
| SEA | 1.14 | 0.55 | 1.35 | 2.15 | 0.83 | 1.04 | 1.45 | 0.60 | 1.29 | 0.99 | 0.72 |
| AJ | 1.13 | 0.46 | 1.24 | 1.86 | 1.05 | 0.91 | 1.18 | 0.71 | 1.03 | 1.06 | 0.87 |
| I | 1.23 | 0.42 | 1.22 | 1.82 | 1.12 | 1.03 | 1.19 | 0.89 | 1.29 | 1.83 | 1.10 |
| FVB | 0.94 | 1.14 | 0.83 | 1.27 | 1.23 | 0.85 | 0.99 | 0.96 | 1.03 | 1.14 | 0.86 |
| MA | 1.00 | 1.00 | 0.91 | 1.44 | 1.14 | 0.77 | 1.05 | 1.53 | 1.05 | 1.01 | 1.20 |
| NON | 1.00 | 0.87 | 0.82 | 1.22 | 1.58 | 0.75 | 1.19 | 1.60 | 0.91 | 0.99 | 1.35 |
