# Supplementary material for: Comparative Analysis of Proteome and Transcriptome Variation in Mouse
Source: PLoS Genet. 2011 Jun 9;7(6):e1001393. doi: 10.1371/journal.pgen.1001393 (PMC3111477; doi:10.1371/journal.pgen.1001393)
Supplement: Table S3 — Biological representation of protein and transcript datasets. (DOC) [file pgen.1001393.s011.doc]

**Table S3.Protein and Transcript Datasets Biological Representation:**

| **GO_Terms** | **GO_ID** | **GO_Class** | **Protein Data** | **Fisher's Exact Test Pvalue for Proteins** | **Transcript Data** | **Fisher's Exact Test Pvalue for Transcripts** | **Background Data (All Annotated Genes by MGI)** |
| --- | --- | --- | --- | --- | --- | --- | --- |
| **mitochondrion** | **GO:0005739** | **Cellular Compartment** | **221 of 477 genes 46.33%** | **6.20E-160** | **892 of 6894 genes 12.94%** | **3.5E-140** | **1436 of 33385 annotated genes 4.30%** |
| **cytoplasm** | **GO:0005737** | **Cellular Compartment** | **196 of 477 genes 41.09%** | **2.75E-41** | **2497 of 6894 genes 36.22%** | **1.3E-300** | **5089 of 33385 annotated genes 15.24%** |
| **endoplasmic reticulum** | **GO:0005783** | **Cellular Compartment** | **72 of 477 genes 15.09%** | **2.17E-28** | **582 of 6894 genes 8.44%** | **5.2E-82** | **998 of 33385 annotated genes 2.99%** |
| **nucleus** | **GO:0005634** | **Cellular Compartment** | **80 of 477 genes 16.77%** | **7.11E-02** | **2220 of 6894 genes 32.20%** | **9.2E-263** | **4610 of 33385 annotated genes 13.81%** |
| **ribosome** | **GO:0005840** | **Cellular Compartment** | **31 of 477 genes 6.50%** | **2.18E-21** | **88 of 6894 genes 1.28%** | **1.3E-08** | **197 of 33385 annotated genes 0.59%** |
| **plasma membrane** | **GO:0005886** | **Cellular Compartment** | **46 of 477 genes 9.64%** | **8.78E-01** | **1115 of 6894 genes 16.17%** | **8.5E-47** | **3321 of 33385 annotated genes 9.95%** |
| **cytosol** | **GO:0005829** | **Cellular Compartment** | **132 of 477 genes 27.67%** | **1.78E-68** | **775 of 6894 genes 11.24%** | **1.1E-109** | **1334 of 33385 annotated genes 4.00%** |
| **extracellular region** | **GO:0005576** | **Cellular Compartment** | **37 of 477 genes 7.76%** | **2.28E-02** | **569 of 6894 genes 8.25%** | **2.1E-20** | **1758 of 33385 annotated genes 5.27%** |
| **peroxisome** | **GO:0005777** | **Cellular Compartment** | **31 of 477 genes 6.50%** | **5.42E-28** | **83 of 6894 genes 1.20%** | **3.0E-17** | **110 of 33385 annotated genes 0.33%** |
| **cytoskeleton** | **GO:0005856** | **Cellular Compartment** | **19 of 477 genes 3.98%** | **9.06E-01** | **538 of 6894 genes 7.80%** | **4.6E-38** | **1325 of 33385 annotated genes 3.97%** |
| **Golgi apparatus** | **GO:0005794** | **Cellular Compartment** | **24 of 477 genes 5.03%** | **3.55E-03** | **460 of 6894 genes 6.67%** | **1.5E-54** | **877 of 33385 annotated genes 2.63%** |
| **vesicle** | **GO:0031982** | **Cellular Compartment** | **18 of 477 genes 3.77%** | **2.33E-02** | **328 of 6894 genes 4.76%** | **3.1E-31** | **705 of 33385 annotated genes 2.11%** |
| **organelle** | **GO:0043226** | **Cellular Compartment** | **12 of 477 genes 2.52%** | **6.34E-01** | **334 of 6894 genes 4.84%** | **9.6E-31** | **731 of 33385 annotated genes 2.19%** |
| **lysosome** | **GO:0005764** | **Cellular Compartment** | **8 of 477 genes 1.68%** | **2.11E-02** | **145 of 6894 genes 2.10%** | **4.0E-23** | **232 of 33385 annotated genes 0.69%** |
| **endosome** | **GO:0005768** | **Cellular Compartment** | **7 of 477 genes 1.47%** | **5.20E-01** | **219 of 6894 genes 3.18%** | **1.3E-28** | **396 of 33385 annotated genes 1.19%** |
| **transport** | **GO:0006810** | **Biological Process** | **107 of 480 genes 22.29%** | **2.05E-19** | **1327 of 6800 genes 19.51%** | **3.0E-136** | **2850 of 33137 annotated genes 8.60%** |
| **biosynthetic process** | **GO:0009058** | **Biological Process** | **119 of 480 genes 24.79%** | **6.48E-51** | **765 of 6800 genes 11.25%** | **4.9E-88** | **1507 of 33137 annotated genes 4.55%** |
| **catabolic process** | **GO:0009056** | **Biological Process** | **140 of 480 genes 29.17%** | **9.82E-71** | **839 of 6800 genes 12.34%** | **3.9E-119** | **1456 of 33137 annotated genes 4.39%** |
| **generation of precursor metabolites and energy** | **GO:0006091** | **Biological Process** | **56 of 480 genes 11.67%** | **2.86E-44** | **156 of 6800 genes 2.29%** | **5.8E-24** | **257 of 33137 annotated genes 0.78%** |
| **lipid metabolic process** | **GO:0006629** | **Biological Process** | **94 of 480 genes 19.58%** | **3.04E-50** | **485 of 6800 genes 7.13%** | **4.2E-64** | **872 of 33137 annotated genes 2.63%** |
| **translation** | **GO:0006412** | **Biological Process** | **57 of 480 genes 11.88%** | **1.73E-34** | **219 of 6800 genes 3.22%** | **4.0E-25** | **433 of 33137 annotated genes 1.31%** |
| **cellular amino acid and derivative metabolic process** | **GO:0006519** | **Biological Process** | **0 of 480 genes 0%** | **1.00E+00** | **0 of 6800 genes 0%** | **1.0E+00** | **0 of 33137 annotated genes 0.00%** |
| **nucleobase, nucleoside, nucleotide and nucleic acid metabolic process** | **GO:0006139** | **Biological Process** | **79 of 480 genes 16.46%** | **7.16E-23** | **776 of 6800 genes 11.41%** | **6.2E-95** | **1476 of 33137 annotated genes 4.45%** |
| **carbohydrate metabolic process** | **GO:0005975** | **Biological Process** | **48 of 480 genes 10.00%** | **1.05E-22** | **282 of 6800 genes 4.15%** | **5.6E-35** | **529 of 33137 annotated genes 1.60%** |
| **multicellular organismal development** | **GO:0007275** | **Biological Process** | **48 of 480 genes 10.00%** | **2.12E-01** | **1087 of 6800 genes 15.99%** | **1.9E-74** | **2768 of 33137 annotated genes 8.35%** |
| **response to stress** | **GO:0006950** | **Biological Process** | **52 of 480 genes 10.83%** | **2.32E-07** | **803 of 6800 genes 11.81%** | **2.8E-87** | **1635 of 33137 annotated genes 4.93%** |
| **protein metabolic process** | **GO:0019538** | **Biological Process** | **36 of 480 genes 7.50%** | **2.35E-06** | **552 of 6800 genes 8.12%** | **1.2E-68** | **1032 of 33137 annotated genes 3.11%** |
| **cell differentiation** | **GO:0030154** | **Biological Process** | **29 of 480 genes 6.04%** | **9.23E-01** | **767 of 6800 genes 11.28%** | **1.3E-49** | **1978 of 33137 annotated genes 5.97%** |
| **signal transduction** | **GO:0007165** | **Biological Process** | **26 of 480 genes 5.42%** | **1.66E-06** | **1044 of 6800 genes 15.35%** | **7.5E-15** | **3926 of 33137 annotated genes 11.85%** |
| **regulation of gene expression** | **GO:0010468** | **Biological Process** | **38 of 480 genes 7.92%** | **8.63E-01** | **1138 of 6800 genes 16.74%** | **5.3E-105** | **2553 of 33137 annotated genes 7.70%** |
| **cellular homeostasis** | **GO:0019725** | **Biological Process** | **28 of 480 genes 5.83%** | **1.14E-08** | **237 of 6800 genes 3.49%** | **1.1E-21** | **528 of 33137 annotated genes 1.59%** |
| **immune system process** | **GO:0002376** | **Biological Process** | **24 of 480 genes 5.00%** | **2.25E-02** | **475 of 6800 genes 6.99%** | **1.6E-46** | **1013 of 33137 annotated genes 3.06%** |
| **response to external stimulus** | **GO:0009605** | **Biological Process** | **17 of 480 genes 3.54%** | **3.41E-02** | **277 of 6800 genes 4.07%** | **4.0E-20** | **683 of 33137 annotated genes 2.06%** |
| **protein modification process** | **GO:0006464** | **Biological Process** | **30 of 480 genes 6.25%** | **4.15E-01** | **897 of 6800 genes 13.19%** | **3.3E-102** | **1787 of 33137 annotated genes 5.39%** |
| **cell death** | **GO:0008219** | **Biological Process** | **35 of 480 genes 7.29%** | **4.38E-04** | **652 of 6800 genes 9.59%** | **6.7E-77** | **1261 of 33137 annotated genes 3.81%** |
| **transcription** | **GO:0006350** | **Biological Process** | **16 of 480 genes 3.33%** | **1.33E-03** | **985 of 6800 genes 14.49%** | **1.1E-83** | **2282 of 33137 annotated genes 6.89%** |
| **cell proliferation** | **GO:0008283** | **Biological Process** | **30 of 480 genes 6.25%** | **5.93E-04** | **488 of 6800 genes 7.18%** | **9.3E-47** | **1052 of 33137 annotated genes 3.17%** |
| **reproduction** | **GO:0000003** | **Biological Process** | **13 of 480 genes 2.71%** | **3.42E-01** | **281 of 6800 genes 4.13%** | **2.2E-19** | **708 of 33137 annotated genes 2.14%** |
| **embryonic development** | **GO:0009790** | **Biological Process** | **14 of 480 genes 2.92%** | **4.47E-01** | **332 of 6800 genes 4.88%** | **2.1E-26** | **785 of 33137 annotated genes 2.37%** |
| **behavior** | **GO:0007610** | **Biological Process** | **9 of 480 genes 1.88%** | **3.09E-01** | **138 of 6800 genes 2.03%** | **2.1E-05** | **439 of 33137 annotated genes 1.32%** |
| **cytoskeleton organization** | **GO:0007010** | **Biological Process** | **7 of 480 genes 1.46%** | **1.00E+00** | **214 of 6800 genes 3.15%** | **1.5E-17** | **504 of 33137 annotated genes 1.52%** |
| **organelle organization** | **GO:0006996** | **Biological Process** | **11 of 480 genes 2.29%** | **6.72E-01** | **477 of 6800 genes 7.01%** | **1.1E-57** | **906 of 33137 annotated genes 2.73%** |
| **growth** | **GO:0040007** | **Biological Process** | **8 of 480 genes 1.67%** | **2.85E-01** | **177 of 6800 genes 2.60%** | **6.3E-17** | **389 of 33137 annotated genes 1.17%** |
| **cell cycle** | **GO:0007049** | **Biological Process** | **12 of 480 genes 2.50%** | **6.83E-01** | **480 of 6800 genes 7.06%** | **4.3E-51** | **979 of 33137 annotated genes 2.95%** |
| **cell-cell signaling** | **GO:0007267** | **Biological Process** | **12 of 480 genes 2.50%** | **2.18E-01** | **177 of 6800 genes 2.60%** | **8.5E-06** | **582 of 33137 annotated genes 1.76%** |
| **DNA metabolic process** | **GO:0006259** | **Biological Process** | **8 of 480 genes 1.67%** | **8.56E-01** | **270 of 6800 genes 3.97%** | **5.5E-30** | **541 of 33137 annotated genes 1.63%** |
| **cell communication** | **GO:0007154** | **Biological Process** | **14 of 480 genes 2.92%** | **8.89E-01** | **431 of 6800 genes 6.34%** | **3.9E-41** | **932 of 33137 annotated genes 2.81%** |
| **mitochondrion organization** | **GO:0007005** | **Biological Process** | **6 of 480 genes 1.25%** | **2.23E-02** | **101 of 6800 genes 1.49%** | **1.1E-18** | **146 of 33137 annotated genes 0.44%** |
| **cell growth** | **GO:0016049** | **Biological Process** | **4 of 480 genes 0.83%** | **7.91E-01** | **122 of 6800 genes 1.79%** | **6.2E-13** | **258 of 33137 annotated genes 0.78%** |
| **regulation of gene expression, epigenetic** | **GO:0040029** | **Biological Process** | **1 of 480 genes 0.21%** | **1.00E+00** | **43 of 6800 genes 0.63%** | **1.2E-05** | **89 of 33137 annotated genes 0.27%** |
| **cell recognition** | **GO:0008037** | **Biological Process** | **0 of 480 genes 0%** | **1.00E+00** | **22 of 6800 genes 0.32%** | **8.9E-03** | **54 of 33137 annotated genes 0.16%** |
| **structural molecule activity** | **GO:0005198** | **Molecular Function** | **29 of 477 genes 6.08%** | **1.98E-12** | **154 of 6729 genes 2.29%** | **6.5E-12** | **380 of 32958 annotated genes 1.15%** |
| **transporter activity** | **GO:0005215** | **Molecular Function** | **38 of 477 genes 7.97%** | **5.39E-07** | **380 of 6729 genes 5.65%** | **5.8E-21** | **1043 of 32958 annotated genes 3.16%** |
| **RNA binding** | **GO:0003723** | **Molecular Function** | **31 of 477 genes 6.50%** | **1.08E-07** | **386 of 6729 genes 5.74%** | **3.6E-50** | **705 of 32958 annotated genes 2.14%** |
| **electron carrier activity** | **GO:0009055** | **Molecular Function** | **26 of 477 genes 5.45%** | **5.76E-22** | **68 of 6729 genes 1.01%** | **1.1E-11** | **109 of 32958 annotated genes 0.33%** |
| **lipid binding** | **GO:0008289** | **Molecular Function** | **41 of 477 genes 8.60%** | **8.86E-22** | **193 of 6729 genes 2.87%** | **7.2E-22** | **388 of 32958 annotated genes 1.18%** |
| **DNA binding** | **GO:0003677** | **Molecular Function** | **10 of 477 genes 2.10%** | **1.11E-03** | **661 of 6729 genes 9.82%** | **7.6E-43** | **1708 of 32958 annotated genes 5.18%** |
| **enzyme regulator activity** | **GO:0030234** | **Molecular Function** | **18 of 477 genes 3.77%** | **4.89E-02** | **335 of 6729 genes 4.98%** | **4.8E-28** | **777 of 32958 annotated genes 2.36%** |
| **kinase activity** | **GO:0016301** | **Molecular Function** | **11 of 477 genes 2.31%** | **7.54E-01** | **355 of 6729 genes 5.28%** | **1.1E-38** | **720 of 32958 annotated genes 2.18%** |
| **antioxidant activity** | **GO:0016209** | **Molecular Function** | **11 of 477 genes 2.31%** | **3.02E-10** | **33 of 6729 genes 0.49%** | **8.5E-08** | **44 of 32958 annotated genes 0.13%** |
| **carbohydrate binding** | **GO:0030246** | **Molecular Function** | **15 of 477 genes 3.14%** | **6.43E-04** | **168 of 6729 genes 2.50%** | **2.6E-15** | **381 of 32958 annotated genes 1.16%** |
| **cytoskeletal protein binding** | **GO:0008092** | **Molecular Function** | **6 of 477 genes 1.26%** | **8.49E-01** | **227 of 6729 genes 3.37%** | **1.6E-22** | **489 of 32958 annotated genes 1.48%** |
| **receptor binding** | **GO:0005102** | **Molecular Function** | **15 of 477 genes 3.14%** | **6.79E-01** | **368 of 6729 genes 5.47%** | **4.8E-24** | **948 of 32958 annotated genes 2.88%** |
| **signal transducer activity** | **GO:0004871** | **Molecular Function** | **8 of 477 genes 1.68%** | **1.79E-10** | **512 of 6729 genes 7.61%** | **4.9E-03** | **2851 of 32958 annotated genes 8.65%** |
| **transcription factor activity** | **GO:0003700** | **Molecular Function** | **1 of 477 genes 0.21%** | **4.46E-04** | **266 of 6729 genes 3.95%** | **1.1E-14** | **736 of 32958 annotated genes 2.23%** |
| **chromatin binding** | **GO:0003682** | **Molecular Function** | **1 of 477 genes 0.21%** | **3.78E-01** | **103 of 6729 genes 1.53%** | **8.7E-13** | **201 of 32958 annotated genes 0.61%** |
| **translation regulator activity** | **GO:0045182** | **Molecular Function** | **2 of 477 genes 0.42%** | **3.89E-02** | **12 of 6729 genes 0.18%** | **7.0E-03** | **20 of 32958 annotated genes 0.06%** |
| **protein kinase activity** | **GO:0004672** | **Molecular Function** | **0 of 477 genes 0%** | **4.87E-04** | **267 of 6729 genes 3.97%** | **1.4E-27** | **560 of 32958 annotated genes 1.70%** |
| **phosphoprotein phosphatase activity** | **GO:0004721** | **Molecular Function** | **0 of 477 genes 0%** | **2.85E-01** | **72 of 6729 genes 1.07%** | **2.8E-08** | **152 of 32958 annotated genes 0.46%** |
