# Supplementary material for: Comparative Analysis of Proteome and Transcriptome Variation in Mouse
Source: PLoS Genet. 2011 Jun 9;7(6):e1001393. doi: 10.1371/journal.pgen.1001393 (PMC3111477; doi:10.1371/journal.pgen.1001393)
Supplement: Table S4 — Correlation analysis of the GO terms for the protein-transcript pairs. (DOC) [file pgen.1001393.s012.doc]

**Table S4. GO Analysis for PT-pairs**

| GO_Name | GO_Class | Number of Genes | Mean Correlation | Mean Correlation Pvalue | Bootstrap Pvalue | Relation |
| --- | --- | --- | --- | --- | --- | --- |
| lipid binding | MF | 36 | 0.30 | 3.32E-04 | 6.27E-02 | - |
| transporter activity | MF | 36 | 0.12 | 1.02E-01 | 4.00E-05 | discordant |
| RNA binding | MF | 24 | 0.08 | 2.31E-01 | <1E-05 | discordant |
| structural molecule activity | MF | 14 | 0.02 | 3.17E-01 | 2.00E-05 | discordant |
| electron carrier activity | MF | 25 | 0.37 | 2.00E-05 | 8.88E-03 | concordant |
| enzyme regulator activity | MF | 17 | 0.29 | 2.90E-03 | 4.60E-01 | - |
| receptor binding | MF | 13 | 0.23 | 9.09E-03 | 6.89E-01 | - |
| carbohydrate binding | MF | 14 | 0.17 | 4.00E-02 | 9.45E-01 | - |
| kinase activity | MF | 9 | 0.30 | 1.36E-03 | 3.23E-01 | - |
| antioxidant activity | MF | 10 | 0.30 | 3.37E-03 | 4.66E-01 | - |
| DNA binding | MF | 7 | 0.13 | 1.29E-01 | 9.68E-01 | - |
| signal transducer activity | MF | 5 | 0.25 | 1.14E-02 | 6.02E-01 | - |
| cytoskeletal protein binding | MF | 6 | 0.19 | 2.84E-02 | 7.75E-01 | - |
| translation regulator activity | MF | 2 | 0.11 | 8.44E-02 | 7.13E-01 | - |
| chromatin binding | MF | 1 | 0.29 | 9.94E-03 | 3.91E-01 | - |
| DNA binding transcription factor activity | MF | 1 | -0.06 | 5.71E-01 | 8.87E-01 | - |
| catabolic process | BP | 124 | 0.27 | 1.35E-03 | 1.33E-01 | - |
| biosynthetic process | BP | 107 | 0.27 | 1.48E-03 | 1.92E-01 | - |
| transport | BP | 96 | 0.16 | 3.71E-02 | <1E-05 | discordant |
| lipid metabolic process | BP | 89 | 0.28 | 1.15E-03 | 1.37E-01 | - |
| nucleic acid metabolic process | BP | 61 | 0.20 | 6.94E-03 | 8.52E-01 | - |
| translation | BP | 23 | 0.03 | 2.76E-01 | <1E-05 | discordant |
| generation of precursor metabolites and energy | BP | 51 | 0.20 | 9.70E-03 | 9.05E-01 | - |
| response to stress | BP | 45 | 0.26 | 3.11E-03 | 5.29E-01 | - |
| multicellular organismal development | BP | 38 | 0.22 | 9.50E-03 | 8.61E-01 | - |
| carbohydrate metabolic process | BP | 44 | 0.28 | 6.05E-04 | 1.03E-01 | - |
| regulation of gene expression | BP | 28 | 0.14 | 6.12E-02 | 3.40E-03 | discordant |
| protein metabolic process | BP | 32 | 0.14 | 8.14E-02 | 3.20E-04 | discordant |
| cell death | BP | 27 | 0.21 | 1.58E-03 | 3.27E-01 | - |
| cell proliferation | BP | 23 | 0.19 | 1.43E-02 | 8.69E-01 | - |
| protein modification process | BP | 24 | 0.21 | 8.65E-03 | 7.67E-01 | - |
| cell differentiation | BP | 19 | 0.23 | 3.90E-03 | 5.36E-01 | - |
| cellular homeostasis | BP | 27 | 0.26 | 5.06E-03 | 6.39E-01 | - |
| signal transduction | BP | 20 | 0.21 | 1.13E-02 | 8.01E-01 | - |
| immune system process | BP | 18 | 0.19 | 3.32E-02 | 9.51E-01 | - |
| response to external stimulus | BP | 14 | 0.25 | 5.19E-04 | 1.95E-01 | - |
| transcription | BP | 11 | 0.15 | 5.45E-02 | 9.45E-01 | - |
| cell communication | BP | 11 | 0.19 | 3.29E-02 | 8.91E-01 | - |
| embryo development | BP | 11 | 0.24 | 7.38E-03 | 6.25E-01 | - |
| reproduction | BP | 10 | 0.30 | 2.24E-04 | 1.47E-01 | - |
| cell-cell signaling | BP | 10 | 0.21 | 8.38E-03 | 6.37E-01 | - |
| cell cycle | BP | 6 | 0.14 | 9.01E-02 | 9.23E-01 | - |
| organelle organization | BP | 6 | 0.07 | 6.06E-02 | 8.76E-01 | - |
| behavior | BP | 8 | 0.26 | 8.81E-03 | 6.20E-01 | - |
| DNA metabolic process | BP | 5 | 0.14 | 9.89E-02 | 9.06E-01 | - |
| growth | BP | 6 | 0.05 | 2.81E-01 | 1.29E-02 | discordant |
| cytoskeleton organization | BP | 6 | 0.19 | 3.60E-02 | 8.10E-01 | - |
| mitochondrion organization | BP | 5 | 0.26 | 2.03E-02 | 6.89E-01 | - |
| cell growth | BP | 3 | 0.04 | 2.63E-01 | 9.41E-01 | - |
| regulation of gene expression epigenetic | BP | 1 | 0.22 | 3.20E-02 | 4.73E-01 | - |
| mitochondrion | CC | 191 | 0.24 | 4.58E-03 | 9.10E-01 | - |
| cytoplasm | CC | 164 | 0.24 | 2.72E-03 | 5.23E-01 | - |
| cytosol | CC | 90 | 0.21 | 8.87E-03 | 9.60E-01 | - |
| nucleus | CC | 60 | 0.19 | 1.29E-02 | 9.69E-01 | - |
| endoplasmic reticulum | CC | 66 | 0.33 | 2.56E-04 | 1.71E-02 | concordant |
| plasma membrane | CC | 36 | 0.17 | 2.90E-02 | 2.24E-02 | discordant |
| extracellular region | CC | 30 | 0.23 | 2.31E-03 | 4.24E-01 | - |
| ribosome | CC | 10 | 0.01 | 3.00E-01 | 6.80E-04 | discordant |
| peroxisome | CC | 27 | 0.32 | 4.59E-04 | 1.23E-01 | - |
| Golgi apparatus | CC | 21 | 0.19 | 1.51E-02 | 8.65E-01 | - |
| cytoskeleton | CC | 17 | 0.12 | 1.08E-01 | 4.04E-03 | discordant |
| vesicle | CC | 11 | 0.18 | 3.65E-02 | 9.04E-01 | - |
| organelle | CC | 10 | 0.18 | 6.95E-02 | 9.57E-01 | - |
| lysosome | CC | 7 | 0.14 | 1.75E-02 | 7.20E-01 | - |
| endosome | CC | 5 | 0.13 | 1.19E-01 | 9.26E-01 | - |
| lipid binding | MF | 36 | 0.30 | 3.32E-04 | 6.27E-02 | - |
