# Supplementary material for: Comparative Analysis of Proteome and Transcriptome Variation in Mouse
Source: PLoS Genet. 2011 Jun 9;7(6):e1001393. doi: 10.1371/journal.pgen.1001393 (PMC3111477; doi:10.1371/journal.pgen.1001393)
Supplement: Table S5 — Effect of SNPs in Affymetrix probes on eQTL detection. (DOC) [file pgen.1001393.s013.doc]

**TableS5. Effect of SNPs in Affymetrix probes on eQTL detection**

| **Total number of probesets** | **Number of probes in each probeset with at least one SNP** | **Number of local eQTLs in the original probesets** | **Number of local eQTLs not detected after removal of probes with SNP** | **Number of new local eQTLs detected after removal of probes with SNP** |
| --- | --- | --- | --- | --- |
| **4636** | **1** | **624** | **14** | **15** |
| **3468** | **2** | **485** | **34** | **20** |
| **2077** | **3** | **352** | **53** | **17** |
| **1188** | **4** | **204** | **45** | **14** |
| **623** | **5** | **109** | **30** | **8** |
| **350** | **6** | **68** | **21** | **3** |
| **205** | **7** | **33** | **14** | **6** |
| **122** | **8** | **20** | ***NA*** | ***NA*** |
| **77** | **9** | **13** | ***NA*** | ***NA*** |
| **52** | **10** | **13** | ***NA*** | ***NA*** |
| **49** | **11** | **8** | ***NA*** | ***NA*** |
