# Supplementary material for: Comparative Analysis of Proteome and Transcriptome Variation in Mouse
Source: PLoS Genet. 2011 Jun 9;7(6):e1001393. doi: 10.1371/journal.pgen.1001393 (PMC3111477; doi:10.1371/journal.pgen.1001393)
Supplement: Table S6 — Effect of biologic replicates on eQTL detection. (DOC) [file pgen.1001393.s014.doc]

**TableS6. Effect of Biologic Replicates on eQTL Detection**

| **Genomewide Significance Cutoff** | **# Local eQTL (3 Mice)** | **# Local eQTL (1 Mouse)** | **# Distant eQTL (3 Mice)** | **# Distant eQTL (1Mouse)** |
| --- | --- | --- | --- | --- |
| **1.00E-06** | **3756** | **2549** | **8267** | **3505** |
| **1.00E-07** | **3333** | **2178** | **4474** | **1782** |
| **1.00E-08** | **2918** | **1846** | **2740** | **1106** |
| **1.00E-09** | **2658** | **1613** | **1953** | **799** |
| **1.00E-10** | **2316** | **1474** | **1469** | **653** |
