# Supplementary material for: Comparative Analysis of Proteome and Transcriptome Variation in Mouse
Source: PLoS Genet. 2011 Jun 9;7(6):e1001393. doi: 10.1371/journal.pgen.1001393 (PMC3111477; doi:10.1371/journal.pgen.1001393)
Supplement: Text S1 — Supplementary material—SNP effect analysis. (DOC) [file pgen.1001393.s015.doc]

**Supplementary Material-SNP Effect Analysis**

To assess the effect of SNPs within the sequence of the oligonucleotide probes designed by Affymetrix, we compared the results of genome-wide association analysis on the original probesets as defined by Affymetrix to the results of genome-wide association performed on Affymetrix probesets masked with probes containing publicly annotated SNPs. Each probeset on the Affymetrix gene expression microarray is comprised of 11 25-mer oligonucleotides. We matched the genomic location of each of these 25-mers to the location of over 8 million SNPs obtained from the Perlegen website (<http://mouse.perlegen.com/mouse/index.html> ) and we flagged any probe which contained one or more SNPs within its sequence. TableS5 shows a summary statistic on the count of SNPs for the AFFY-HT430A chip.

To assess the effect of SNP on association results, we performed genome-wide association analysis on two datasets. The first dataset comprised of the probesets as defined by affymetrix (each probeset contains all the 11 probes selected by affymetrix). The second dataset comprised of probesets containing all probes selected by Affymetrix except for those 25-mer probes flagged for containing one or more SNPs. As a result the second dataset contained probesets each having varying number of probes. If SNPs have a minimal/no effect on eQTL discovery, one would expect to have both a similar number of counts and for local eQTLs obtained from each dataset. The last two columns in TableS5 show that removal probes with SNP results in both reduction in number of eQTLs and detection of new local eQTLs. Based on these results, we removed all the probes within each probeset before estimating the expression level of each probeset.

**Effect of Replicates on eQTL Detection**

Transcript levels in inbred stains were measured by profiling three mice for each strain, using Affymetrix MOE430A platform, and taking the average of expression over the three biological replicates. One advantage of this design is to estimate the transcript levels more accurately for each inbred strain. In order to investigate the effect of using replicates on eQTL detection, we randomly chose one mouse per strain and perform genome-wide association on this dataset. We compared the results of this genome-wide study to the mapping results obtained from dataset comprising the averaged values. TableS6 shows the count of local and distant eQTL in each study.

These results indicate, as expected, that the use of replicates provides better power to detect both local and distant eQTLs. Averaging over various genome-wide cutoffs tested, there are 36% less Local and 58% less Distant eQTLs detected when data from one mouse is used as compared to using average of three mice per strain.
